# Transcriptional repression of TGFB2-AS1 by GATA6 drives triple-negative breast cancer metastasis

**DOI:** 10.1007/s13402-026-01195-5

**Published:** 2026-04-01

**Authors:** Chang Liu, Qianru Yu, Difei Wang, Zheng Duan, Xin Zhang, Jiao Wang, Xiaoyu Qi, Jiayin Ye, Qian Zhao, Jianrong He, Cixiang Zhou

**Affiliations:** 1 Institute for Translational Medicine on Cell Fate and Disease, Shanghai Ninth People’s Hospital, Key Laboratory of Cell Differentiation and Apoptosis of National Ministry of Education, Department of Pathophysiology, Shanghai Jiao Tong University School of Medicine, 201318 Shanghai, China; 2 Shanghai-MOST Key Laboratory of Health and Disease Genomics, NHC Key Lab of Reproduction Regulation, Shanghai Engineering Research Center of Reproductive Health Drug and Devices, Shanghai Institute for Biomedical and Pharmaceutical Technologies, Pharmacy School, Fudan University, 200032 Shanghai, China; 3 Department of General Surgery, Comprehensive Breast Health Center, Ruijin Hospital, SJTU-SM, 200025 Shanghai, China

**Keywords:** TGFB2-AS1, GATA6, Triple-negative breast cancer, Metastasis, Diagnosis and treatment strategies

## Abstract

**Purpose:**

High recurrence rates, significant metastatic potential, and limited overall survival make triple-negative breast cancer (TNBC) the most challenging subtype among breast cancers. Previous studies have indicated that the downregulation of TGFB2-AS1 can enhance the stem-like properties of tumor cells, thereby promoting TNBC progression. Bioinformatics analysis has revealed the regulatory role of GATA6 in TGFB2-AS1 transcription, providing insights into the transcriptional regulation of TGFB2-AS1 by GATA6 and offering potential prognostic biomarkers and therapeutic strategies for TNBC.

**Methods:**

Bioinformatics analysis, Western blot, and qPCR were employed to assess the expression of GATA6 and TGFB2-AS1. Immunohistochemistry (IHC) and RNA in situ hybridization (ISH) were performed on clinical samples to evaluate GATA6 and TGFB2-AS1 expression, respectively, with survival analysis based on follow-up data. Fluorescence in situ hybridization (FISH), chromatin immunoprecipitation (ChIP), and dual-luciferase reporter assays were used to elucidate the regulatory mechanisms of GATA6 on TGFB2-AS1. Functional experiments, Western blotting, qPCR, and tail vein metastasis assays, were conducted to investigate the role of GATA6-regulated TGFB2-AS1 in TNBC.

**Results:**

GATA6 binds to the TGFB2-AS1 promoter and represses its transcription, and patients with tumors exhibiting high GATA6 and low TGFB2-AS1 expression are associated with poor prognosis. Both in vivo and in vitro functional experiments confirmed that TGFB2-AS1 critically mediates the tumor-promoting effects of GATA6 in TNBC progression.

**Conclusions:**

Our findings reveal that GATA6 drives the progression of TNBC by repressing TGFB2-AS1 transcription.

**Supplementary Information:**

The online version contains supplementary material available at 10.1007/s13402-026-01195-5.

## Introduction

Breast cancer remains the most prevalent cancer among women, accounting for 32% of all newly diagnosed cancer cases and responsible for approximately 14% of cancer-related deaths in 2024, as estimated [1]. Among all breast cancer subtypes, ‌TNBC constitutes 10% to 20% of cases and is considered the most challenging subtype due to its aggressive clinical course, rapid progression, high rate of early recurrence, and strong propensity for distant metastasis-even when diagnosed at an early stage. TNBC is characterized by the simultaneous absence of estrogen receptor (ER), progesterone receptor (PR), and human epidermal growth factor receptor 2 (HER2) expression. Which renders patient’s ineligible for endocrine therapy or anti-HER2 monoclonal antibody treatment [1, 2].

GATA6 belongs to a small family of zinc-finger transcription factors that are expressed during early embryogenesis and play crucial roles in the development of the gut, lung, liver, heart, and adipose tissue. Mutations in GATA6 are association with congenital abnormalities in multiple organs [3–7]. Notably, GATA6 is frequently upregulated in various cancers and contributes to cancer initiation and progression [8, 9]. However, its role in cancer is context-dependent. For example, in pancreatic ductal carcinoma (PDAC) and lung adenocarcinoma, GATA6 maintains the epithelioid phenotype and suppresses epithelial-mesenchymal transition (EMT), thereby inhibiting cancer cell dissemination [8, 10, 11]. In contrast, in breast cancer, GATA6 promotes EMT and metastatic spread [12], highlighting its tissue-specific regulatory duality.

Long non-coding RNAs (lncRNAs) are typically large, structurally flexible molecules that participate in diverse regulatory processes chromatin organization, transcriptional control, formation of biomolecular condensates, and acting as decoys for proteins and other RNAs through RNA-protein, RNA-DNA, and RNA-RNA interactions [13–16]. Over the past decades, numerous rigorous studies have firmly established that LncRNAs occupy a pivotal and irreplaceable role in tumor progression [17–19]. Our previous work demonstrated that TGFB2-AS1, a metastasis-suppressive lncRNA, interacts with SMARCA4 to transcriptionally repress TGFβ2 and SOX2, thereby attenuating breast cancer stem-like cell (BCSC) properties and lung metastasis in TNBC models [20]. However, the upstream regulatory mechanisms regulating TGFB2-AS1 expression remain unclear. In this study, we identify GATA6 as a direct transcriptional repressor of TGFB2-AS1 through binding its promoter, thereby establishing a critical role for GATA6 in driving TNBC metastasis.

## Materials and methods

### Clinical specimen

119 TNBC samples were obtained from the Comprehensive Breast Health Center, Shanghai Rui-Jin Hospital, Shanghai Jiao Tong University School of Medicine, China. Patients were fully informed and consented. The study was approved by the Ethics Committee of Shanghai Jiao Tong University School of Medicine. The expression of TGFB2-AS1 and GATA6 was detected by in situ hybridization (ISH) and immunohistochemistry (IHC), and the correlation with prognosis was analyzed are shown in Supplementary Table 1.

### Cell lines and cell culture

MDA-MB-231 and MDA-MB-468 were cultured in Leibovitz L-15, BT-549, SUM149PT and HCC1937 in RPMI 1640, LM2 and HEK293T in high glucose DMEM, and all with 10% FBS. SUM159PT used Ham’s F-12 supplemented with 5% FBS, 10 mM HEPES, 1 µg/ml Hydrocortisone and 5 µg/ml Insulin. Cells maintained at 37 °C, 5% CO₂, 95% air except for MDA-MB-231 and MDA-MB-468 in 100% air. Short tandem repeat (STR) profiling confirmed cell identity.

### Bioinformatics prediction of transcription factor binding sites

To identify potential transcription factors (TFs) regulating TGFB2-AS1, we analyzed the 3-kb genomic region upstream of the TGFB2-AS1 transcription start site (TSS) based on the human reference genome assembly GRCh38/hg38. In silico prediction of transcription factor binding sites (TFBSs) was performed using two position weight matrix (PWM)-based databases: JASPAR 2022 (https://jaspar.genereg.net/) and PROMO v3.0 (http://alggen.lsi.upc.es/). These tools were used to scan the promoter sequence for putative TF–DNA interactions. In parallel, experimentally supported TF-target interactions were retrieved from two ChIP-seq-derived resources: the Gene Transcription Regulation Database (GTRD; http://gtrd.biouml.org/) and AnimalTFDB v4.0 (http://bioinfo.life.hust.edu.cn/AnimalTFDB/), specifically its human TF-target compendium (hTFtarget). Both databases provide curated evidence of in vivo TF binding events across human cell types and conditions. The final set of candidate transcriptional regulators was defined by intersecting the outputs from all four resources, thereby integrating computational predictions with empirical binding evidence, to yield seven high-confidence TFs potentially involved in the regulation of TGFB2-AS1.

### Ingenuity pathways analysis (IPA)

The Ingenuity pathway analysis (IPA, Qiagen, Redwood City, http://www.ingenuity.com/) tool was used for pathway analysis and upstream regulator prediction. First, we uploaded gene information, fold-change values, and log-transformed p-values of the differentially expressed genes (DEGs) into the software. Following data upload, a core analysis was performed with the expression analysis, using a screening threshold of |log^FC^| > 1.5 in expression.

### Correlation analysis between GATA6 expression and clinicopathological characteristics

This retrospective study analyzed 119 TNBC samples from Shanghai Rui-Jin Hospital in Supplementary Table 1. GATA6 protein expression in tumor tissues was detected using immunohistochemistry, and patients were categorized into high and low GATA6 expression groups based on scoring results. To evaluate the correlation between GATA6 expression levels and clinicopathological features—including menopausal status, histological grade, TNM stage, history of breast disease, age, and lymph node metastasis—Pearson’s chi-square (χ²) test was employed for statistical analysis.

### Multivariable cox regression analysis

70 cases with clear expression profiles were selected for survival analysis from Shanghai Rui-Jin Hospital in Supplementary Tables 1—36 in the GATA6^high^/TGFB2-AS1^low^ group and 34 in the GATA6^low^/TGFB2-AS1^high^ group. Multivariable Cox regression was performed to compare survival outcomes between the two groups, with adjustment for clinicopathological factors including TNM stage and history of breast cancer. Separate models were fitted for disease-free survival (DFS) and overall survival (OS). Results are presented as hazard ratios (HR) with 95% confidence intervals (CI). All analyses were conducted using SPSS software, with a two-sided P-value < 0.05 considered statistically significant.

### Generated a GATA6-inducible cell line (pINDUCER20-GATA6): plasmid construction, lentiviral production, infection, stabilized cell line selection, and Doxycycline induction conditions

The Homo sapiens GATA6 sequence was amplified by PCR, then inserted into the digested pENTR1A vector via BP recombination reaction, followed by transferring the GATA6 sequence into the pINDUCER20 vector via LR recombination reaction according to the Gateway Technology Manual (Catalog nos. 12535-019 and 12535-027).Virus Production: Plasmids pSPAX-2, pMD2.G and pINDUCER20-GATA6 were transfected into 293T cells at a ratio of 5:3:2 using Lipofectamine 2000 (TL201, Novozymes) for lentivirus production. Stabilized Modified Cell Construction: MDA-231 cells were infected with the viral supernatant supplemented with polybrene. Positive cells were selected with 0.4 µg/ml puromycin for 3 days. Induction Conditions: cells were subsequently treated with 1 ng/µl Doxycycline (Dox) for 4 days.

### Cell transfection and small interfering RNA (siRNA) transfection

The characters of siRNA and plasmids are shown in Supplementary Table 2. siRNAs were purchased from Shanghai GenePharma Co. Ltd and plasmids were generated by our lab. siRNAs transfection (100 nM) was performed using lipomaster 2000 according to the reverse transfection protocol. For plasmid transfection, the ratio of DNA (µg): lipomaster 2000 (µl) was 1:2.5, plasmids and reagents were diluted with Leibovitz L-15. DNA-liposome complex formation and cell transfection were performed according to the lipomaster 2000 manual.

### RNA extraction, reverse transcription PCR and quantitative real-time PCR

Total RNA was extracted using TRIzol reagent (Vazyme) acting as the manual, and first-strand cDNA was synthesised using a reverse transcriptase kit (Vazyme). Quantitative real-time PCR (qPCR) as conducted utilizing SYBR Green (Vazyme) method on a 7900 Real-Time PCR System using the SDS 2.4 software sequence detection system (Applied Biosystems, USA). Gene mRNA levels were quantified using β-actin as an internal control. Relative levels of RNA were calculated using the comparative CT method (2-ΔΔCT). The sequences of the gene-specific primers are listed in Supplementary Table 2.

### Cytoplasmic and nuclear RNA extraction

Cytoplasmic and nuclear RNA extraction and separation were performed using the Cytoplasmic/Nuclear RNA Rapid Extraction Kit (Product No. PS1544). Cells or tissue samples were lysed with pre-cooled Buffer RLN and centrifuged at 13,000 rpm for 3 min to separate the supernatant (cytoplasmic RNA) and pellet (nuclear RNA). The cytoplasmic RNA fraction was processed with RLT Plus lysis buffer and ethanol, then purified using the RA adsorption column, followed by sequential washing with RW1 deproteinization solution and RW wash buffer before final elution. The nuclear RNA fraction was resuspended in RLT Plus, loaded onto the column to collect the filtrate, mixed with ethanol, and purified again on a fresh column, with subsequent washing and elution steps performed as described for cytoplasmic RNA extraction. All procedures were carried out at room temperature using fresh samples and in accordance with reagent safety protocols.

### In vivo assays

Animal Model: four-week-old female BALB/c nude mice were purchased and acclimatized for 2 weeks. Lung Metastasis Experiment: a suspension of 3 × 10⁵ cells in 1×PBS was injected into the tail vein. Bioluminescence imaging (BLI) was monitored using the IVIS imaging system after intraperitoneal injection of D-luciferin potassium salt substrate (200 µl of 15 mg/ml). BLI was monitored weekly starting from the second week, and mice were sacrificed after 4 weeks. Lungs were dissected and fixed in 4% paraformaldehyde.

### Transwell migration and invasion assays

Cell migration and invasion ability were determined by Corning transwell insert chambers (8 μm pore size; Corning) without or with Matrigel paved (Corning BioCoat), respectively. The chemoattractant was 500 ml or 600 ml medium containing 10% FBS which were added into the lower well of each chamber. The prepared cells (1.5 × 10^4^ for migration, 3 × 10^4^ for invasion) were added to the chamber and incubated at 37 °C for 22 h.

### Soft agar colony formation assay, plate colony formation assay and cell proliferation assay

Soft Agar Colony Formation Assay: 3.5 cm dishes were coated with 1.5 ml of 0.8% low-melt agar. After solidification, 0.4% low-melt agar containing 1.5 × 10⁴ cells was added and incubated at 37 °C for two months. The number of colonies with a diameter greater than 50 μm was counted for quantitative analysis. Plate Colony Formation Assay: 800 cells of LM2 cell line were inoculated into 3.5 cm dishes and 1000 cells of MDA-231 cell line were inoculated into separate 3.5 cm dishes. Both were cultured for 12 days with medium changes every 3 days. Colonies were stained with crystal violet (Beyotime) and counted under a microscope, colonies with a diameter greater than 0.5 mm were counted under a microscope. Cell Proliferation Assay (CCK-8): cells were diluted to different concentrations (3.6 × 10⁵, 2.4 × 10⁵, 1.2 × 10⁵, 6 × 10⁴, 3 × 10⁴ cells/ml) and 100 µl was added to each well. After 5 h of incubation, 10 µl of CCK-8 was added. After 2 h, absorbance at 450 nm was measured to plot a standard curve. Each well was inoculated with 3000 cells, and absorbance was measured at 24, 48, and 72 h to plot a growth curve.

### ChIP-qPCR

In brief, 4 × 10^6^ cells requestioned per reaction. Cross-linking was performed in growth medium with 1% formaldehyde for 10 min at RT, and quenched lasting 5 min using 0.125 M glycine at RT. Cells were lysed by ChIP lysis buffer (CST), treated with 0.5 µl Micrococcal Nuclease (CST, #10011), and sonicated to yield DNA fragment sizes of 0.2 to 1 kb. Keeping 10% of the sample as input DNA, the remaining samples with 10 µg GATA6 antibody (proteintech, #55435-1-AP), SMARCA4 antibody (CST, #12483T) or rabbit normal immunoglobulin (IgG) incubated overnight at 4 °C on a rotator. Next day, ChIP-Grade Protein G Magnetic Beads (CST, #9006) were used to precipitate the chromatin. The chromatin was eluted and cross-linking reversed at 65℃. ChIP DNA was quantified by qPCR. The primers used are listed in Supplementary Table 2.

### Immunoblotting

Total proteins abstracted using 1× SDS lysis buffer were separated by SDS-PAGE and transferred to nitrocellulose membranes (Oxygen, CA). After blocking with 5% skim milk, the membranes were incubated overnight at 4 °C with primary antibody followed by horseradish peroxide-coupled secondary antibody. Signals were detected in a sensitive digital imaging device (ImageQuant LAS 4000 mini, GE, USA) using an ECL detection kit (Millipore).

### Dual luciferase reporter assay

Cells were co-transfected with the PGL4.27 plasmid containing the upstream promoter region of TGFB2-AS1 and Renilla as an internal reference, and the expression plasmid of pcDNA3.1-GATA6, and the cells were lysed with 1×PLB (promega) after 48 h and centrifuged at 12,000 g, 10 min, at 4℃. The supernatant was used to determine enzyme activity, whereas the precipitate concentrated nuclear protein, which can be used for GATA6 expression.

### RNA FISH, RNA ISH and IHC analysis

Probe Design: LNA probes (Exiqon) were used, designed with Primer 3 and optimized using Exiqon’s LNA Oligo Optimizer. Specificity was verified by BLAST. RNA FISH: cell-coated slides were fixed, treated, dehydrated, hybridized with DIG-labeled probes, and washed. RNA ISH: paraffin-embedded tissue slides were dewaxed, rehydrated, treated with pepsin, hybridized with AP-conjugated anti-DIG antibodies, and stained with BCIP/NBT. IHC: paraffin-embedded tissue slides were deparaffinized, rehydrated, treated with hydrogen peroxide, antigen retrieval was performed, and slides were blocked and incubated with primary and secondary antibodies. Staining was visualized with DAB and counterstained with hematoxylin.

### TCPA database and TCGA database download

GATA6 protein expression data for BRCA were obtained from the TCPA database (https://tcpaportal.org/tcpa/index.html). RNA sequencing data and corresponding clinical information for BRCA were downloaded from Xena (https://xena.ucsc.edu/). Normalized GATA6 protein levels were matched with AS1 RNA expression and clinical data using the VLOOKUP function in Excel, followed by correlation analysis and Kaplan–Meier survival analysis.

### Statistical analysis

Data from three independent experiments were normalized as follows: the mean value of the control group was calculated first, and data from both the control and treatment groups were normalized to this mean. For both qPCR and protein data from the three experiments, the control group in each experiment was set as 1, and the experimental groups were compared relative to their respective controls. Statistical analysis was performed using GraphPad Prism 8. Differences between groups were assessed using a two-tailed unpaired Student’s t-test, with significance levels denoted as follows: *P < 0.05, **P < 0.01, and ***P < 0.001.

## Results

### GATA6 is a candidate transcription repressor of TGFB2-AS1

Previous studies have shown that TGFB2-AS1 is significantly downregulated in the highly metastatic subclass cell line MDA-MB-231-LM2 (abbreviated as LM2; luciferase-expressing) compared with its parental line, MDA-MB-231 (abbreviated as MDA-231). This downregulation is associated with a poor prognosis in patients TNBC [20]. To identify transcriptional regulators of TGFB2-AS1, we employed two complementary bioinformatics approaches. First, we integrated predictions from four complementary TF databases (JASPAR, PROMO, GTRD, and AnimalTFDB) [21–24], yielding seven high-confidence candidate transcription factors (listed in Supplementary Table 3). Second, we incorporated the top 67 upstream regulators predicted by Ingenuity Pathway Analysis (IPA). Notably, IPA was performed using a high-confidence subset of differentially expressed genes (DEGs) derived from microarray data comprising LM2 and MDA-231 cells [20, 21]. While the full dataset comprised 12,643 DEGs, only those with an absolute fold change > 1.5 were used as input for IPA, resulting in 366 high-confidence DEGs (listed in Supplementary Table 3). The intersection of these two approaches identified GATA6, YY1, and SP1 as candidate regulators (Supplementary Fig. 1A).

To prioritize candidates for functional validation, we assessed their clinical relevance using transcriptomic and survival data from The Cancer Genome Atlas (TCGA) breast invasive carcinoma (BRCA) cohort, accessed via the UCSC Xena platform (https://xena.ucsc.edu/). Kaplan-Meier analysis revealed that high GATA6 expression was significantly associated with worse 5-year overall survival in breast cancer patients, whereas YY1 or SP1 expression showed no significant prognostic value (Supplementary Fig. 1B-D; Supplementary Table 4). Bioinformatic analysis of The Cancer Proteome Atlas (TCPA) data—a proteogenomic dataset— further indicated that GATA6 protein expression was highest in the basal-like subtype among breast cancer molecular subtypes. Although no statistically significant difference was observed between basal-like tumors and normal-like tissues (Supplementary Fig. 1E). Notably, elevated GATA6 levels were associated with larger primary tumors (T3 vs. T1/T2; *P* < 0.05) (Supplementary Fig. 1F), and an increased risk of lung metastasis (*P* < 0.01) (Supplementary Fig. 1G). Given that basal-like tumors constitute the majority of TNBC cases, these findings implicate GATA6 as a candidate regulator of aggressive disease features in TNBC—prompting us to investigate its potential transcriptional regulation of TGFB2-AS1.

**Fig. 1:**
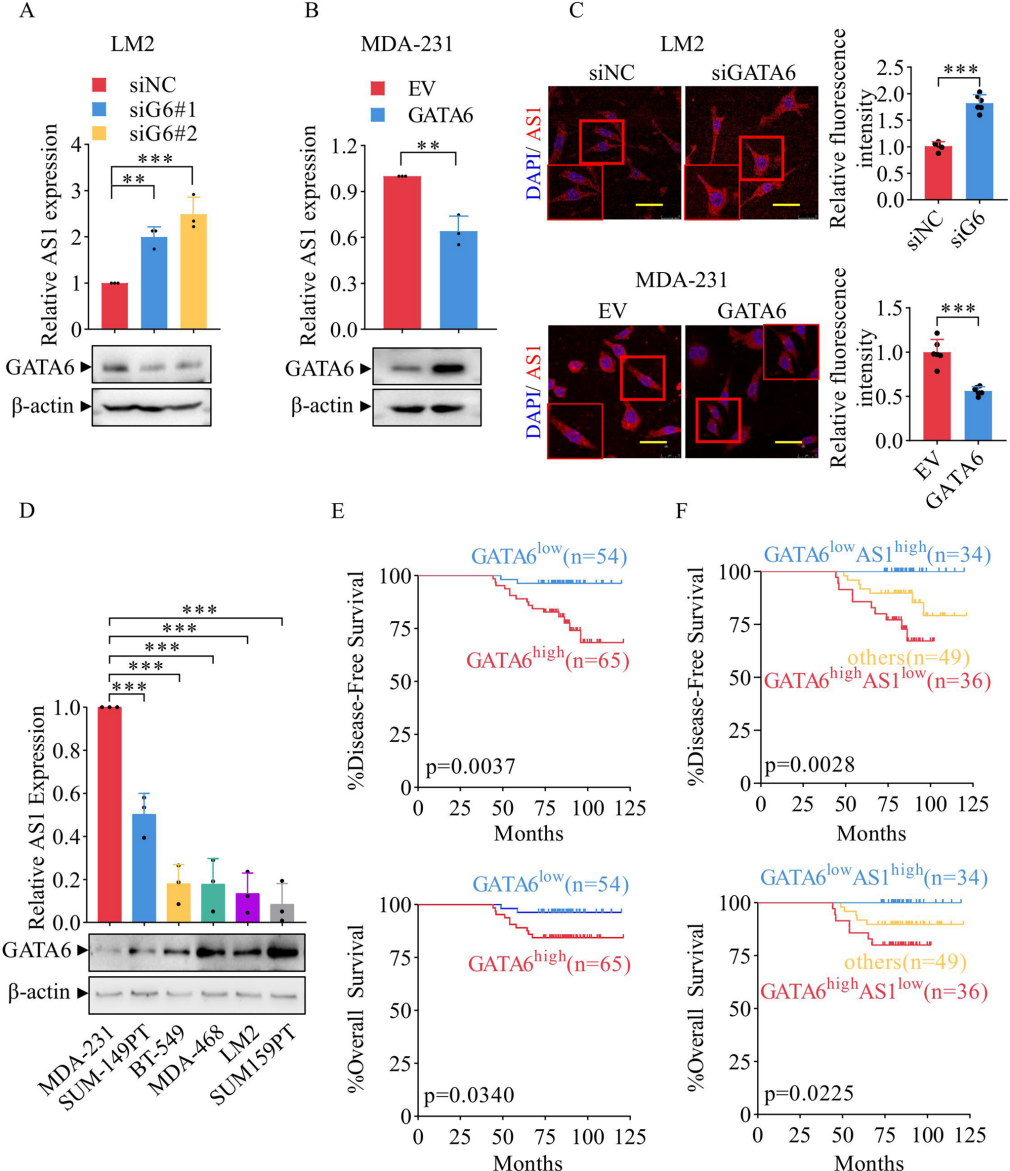
GATA6 is the candidate transcription repressor for TGFB2-AS1 and correlate positively with malignancy of TNBC. (**A**) TGFB2-AS1 (short name: AS1) expression is upregulated upon GATA6 knockdown, as confirmed by qPCR (up) and Western blotting (down). (**B**) AS1 expression is inhibited upon GATA6 overexpression, as confirmed by qPCR (up) and Western blotting (down). (**C**) The images show FISH detection of AS1 in LM2 cells transfected with siRNA (up) and in MDA-231 cells transfected with the indicated plasmids (down). Red fluorescence indicates TGFB2-AS1, and blue fluorescence indicates DAPI (scale: 50 μm). (**D**) Detection of TGFB2-AS1 (up) using qPCR and GATA6 expression (down) using Western blotting in multiple TNBC cell lines. (**E**) The correlation between GATA6 expression levels and survival outcomes in a cohort of 119 TNBC cases, including DFS (up) and OS (down). (**F**) The relationships between the expression of GATA6/TGFB2-AS1 and survival outcomes in a cohort of 119 TNBC cases, including DFS (up) and OS (down)

### Concurrent high GATA6 and low TGFB2-AS1 expression predicts aggressive TNBC phenotype

To identify the transcription factor responsible for TGFB2-AS1 regulation, MDA-231 cells were transfected with siRNAs targeting YY1 or SP1. qPCR analysis showed no significant change in TGFB2-AS1 expression upon efficient knockdown of either SP1 (Supplementary Fig. 2A) or YY1 (Supplementary Fig. 2B). In contrast, a robust inverse correlation between GATA6 and TGFB2-AS1 was observed: TGFB2-AS1 expression was upregulated following GATA6 knockdown in LM2 cells (siGATA6#1, siGATA6#2; Fig. 1A) and significantly downregulated upon ectopic overexpression of GATA6 in MDA-231 cells (Fig. 1B). This reciprocal relationship was further confirmed by FISH for TGFB2-AS1 (Fig. 1C). Notably, the negative correlation between GATA6 and TGFB2-AS1 was conserved across multiple TNBC cell lines. Higher GATA6 and lower TGFB2-AS1 levels were consistently observed in more aggressive TNBC cell lines, including LM2 versus MDA-231 and SUM159PT versus SUM149PT (Fig. 1D; Supplementary Fig. 2C-E). Collectively, these findings establish GATA6 as the predominant transcriptional repressor of TGFB2-AS1 in TNBC.

The clinical relevance of the inverse correlation between GATA6 and TGFB2-AS1 was investigated using a TNBC tissue microarray (119 samples from Ruijin Hospital) (Supplementary Table 1). GATA6 protein was assessed by IHC, while TGFB2-AS1 RNA was detected by ISH with locked nucleic acid (LNA) probes. To evaluate the clinicopathological significance of GATA6, we performed Pearson’s chi-squared tests and found that high GATA6 expression was significantly associated with more advanced TNM stage (Stage I vs. Stage II–III; *P* = 0.042), but not with histological grade (*P* = 0.420), lymph node metastasis status (*P* = 0.781), menopausal status (*P* = 0.061), age (*P* = 0.095), or history of breast disease (*P* = 0.86) (Supplementary Table 5).

Kaplan–Meier analysis revealed that high GATA6 expression was associated with shorter disease-free survival (DFS) and overall survival (OS) (Fig. 1E). Strikingly, patients harboring the combined GATA6high/TGFB2-AS1low expression profile exhibited the worst DFS and OS compared to those with the reciprocal GATA6low/TGFB2-AS1high profile (Fig. 1F).

However, in multivariable Cox regression models adjusted for TNM stage and history of breast disease, the GATA6high/TGFB2-AS1low signature did not reach statistical significance as an independent prognostic factor—though it showed a trend toward worse DFS (HR = 1.86, 95% CI: 0.88–3.93; *P* = 0.104) and no significant association with OS (HR = 1.15, 95% CI: 0.39–3.37; *P* = 0.799). Notably, advanced TNM stage was independently associated with worse OS (HR = 3.21, 95% CI: 1.06–9.71; *P* = 0.039), but not with DFS (*P* = 0.287) (Supplementary Fig. 2F–G). The lack of statistical significance in multivariable models may reflect limited power due to sample size.

To explore the biological basis underlying this prognostic pattern, we examined the correlation between GATA6 and TGFB2-AS1 expression across the entire cohort (*n* = 119). No significant linear correlation was observed overall (Pearson’s *r* = − 0.126, R² = 0.0159, *P* = 0.172; Supplementary Fig. 2H), likely reflecting the known heterogeneity of TNBC. We therefore focused on the two extreme subgroups defined a priori by IHC/ISH scoring: GATA6high/TGFB2-AS1low (*n* = 36) and GATA6low/TGFB2-AS1high (*n* = 34)—together comprising 70 patients who displayed the most divergent survival outcomes (Fig. 1F). In this clinically relevant subset, GATA6 and TGFB2-AS1 exhibited the inverse expression relationship. In contrast, the remaining 49 patients (“Others”—exhibiting concordant high/high or low/low expression) showed intermediate survival outcomes positioned between the two extremes, further underscoring the biological heterogeneity of TNBC and suggesting distinct regulatory contexts.

These clinical observations, together with our experimental evidence demonstrating GATA6-mediated transcriptional repression of TGFB2-AS1, support a model in which their inverse expression relationship contributes to TNBC aggressiveness—particularly in tumors exhibiting this discordant signature—and warrant further validation in larger prospective cohorts.

### GATA6 directly binds the TGFB2-AS1 promoter to suppress transcription

**Fig. 2:**
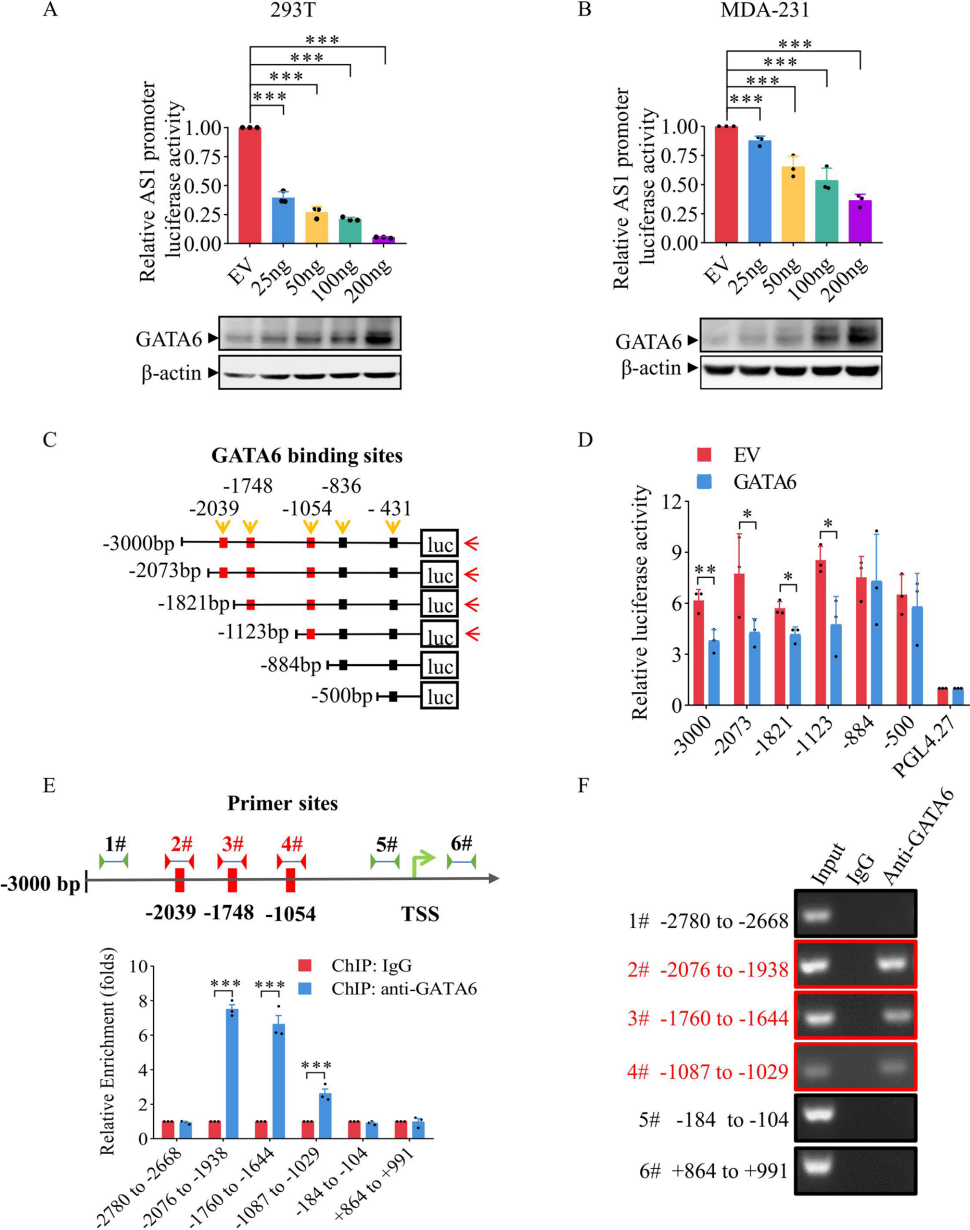
GATA6 binds directly to TGFB2-AS1’s promoter, suppressing its transcription. (**A**) The effect of TGFB2-AS1 promoter luciferase activity (up) and the efficiency of GATA6 expression by WB (down) on different doses of exogenous GATA6 in 293T cells. (**B**) The effect of TGFB2-AS1 promoter luciferase activity (up) and the efficiency of GATA6 expression by WB (down) on different doses of exogenous GATA6 in MDA-231 cells. (**C**) Graphical representation of plasmids with different truncate of AS1 promoter. (**D**) Assaying the luciferase activity of different truncate of AS1 promoter with exogenous GATA6. (**E**) Graphical representation of primer sites using in ChIP-qPCR (up); the enrichment of different TGFB2-AS1 promoter regions by anti-GATA6 comparing to normal IgG (down). (**F**) The pictures of Agarose Gel Electrophoresis of ChIP-qPCR products

To elucidate GATA6-mediated transcriptional repression, the full-length TGFB2-AS1 promoter (-3000 to + 0 bp) was cloned into a luciferase reporter plasmid (pGL4.27-AS1-FL). In 293T and MDA-231 cells transfected with GATA6, luciferase activity was dose-dependently reduced by exogenous GATA6 (Fig. 2A-B). To dissect the underlying mechanism, five putative GATA6 binding motifs [(A/T)GATA(A/G)] were predicted within the TGFB2-AS1 promoter using JASPAR 2022 (Supplementary Table 6), and serial truncations were generated to delete specific motifs (T1-T5; Fig. 2C). Luciferase assays in GATA6-expressing MDA-231 cells demonstrated that retention of the sites at -836/-824 and − 431/-419 (T1-T2 truncations) did not significantly alter promoter activity, whereas retention of the sites at -2039-2027, -1748-1736, and − 1054 − 1042 (T3-T5 truncations) resulted in significantly repression (Fig. 2D). Furthermore, ChIP was performed using GATA6 antibody, followed by qPCR with primers spanning the predicted binding sites. DNA fragments containing − 2039/-2027, -1748/-1736, and − 1054/-1042 motifs were specifically enriched compared to negative control regions (Fig. 2E-F). These data confirm that GATA6 directly binds three distal sites in the TGFB2-AS1 promoter to repress its transcription.

### Knockdown of GATA6 suppresses TNBC malignancy through TGFB2-AS1

**Fig. 3:**
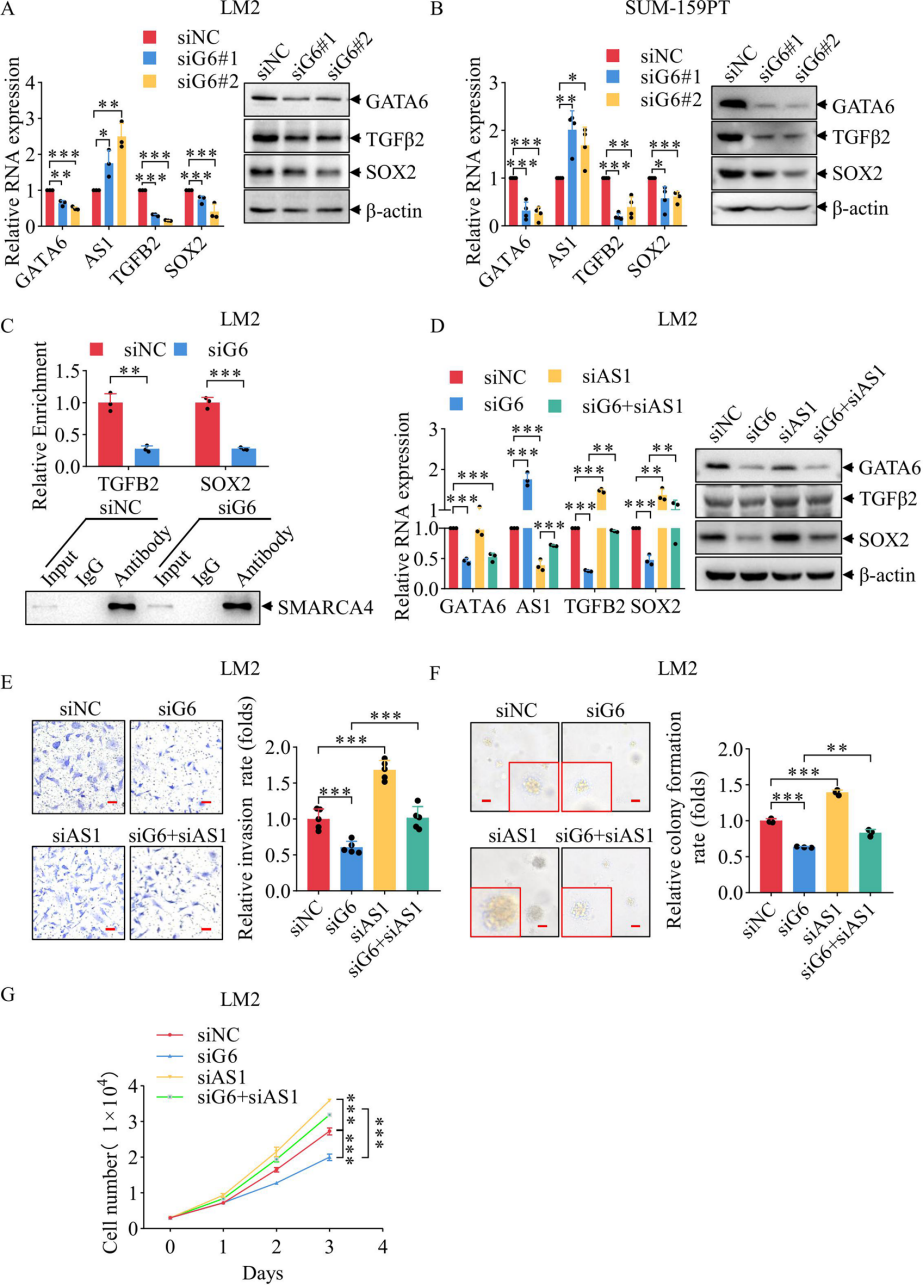
Knockdown of GATA6 suppresses TNBC malignancy through TGFB2-AS1. (**A**) The RNA (left) and protein (right) expression levels of GATA6, TGFB2-AS1, TGFβ2, and SOX2 after siGATA6 transfection in LM2 cells with β-actin as the internal reference. (**B**) The RNA (left) and protein (right) expression levels of GATA6, TGFB2-AS1, TGFβ2, and SOX2 after siGATA6 transfection in SUM159PT cells with β-actin as the internal reference. (**C**) Enrichment of the TGFB2 and SOX2 promoter regions was assessed by ChIP-qPCR using an anti-SMARCA4 antibody (up) and using the same antibody to verify immunoprecipitation efficiency by WB (down) after siGATA6 transfection in LM2 cells. (**D**) The RNA (left) and protein (right) expression levels of GATA6, TGFB2-AS1, TGFβ2, and SOX2 following individual knockdown of GATA6 and TGFB2-AS1, as well as simultaneous knockdown of both genes in LM2 cells with β-actin as the internal reference. (**E**) Illustrative photographs (left, scale: 50 μm) showing the invasion capacity of LM2 cells following individual knockdown of GATA6 and TGFB2-AS1, as well as simultaneous knockdown of both genes, and corresponding statistical analysis (right) of these invasion assays. (**F**) Representative images (left, scale: 50 μm) showing the soft agar colony formation capacity of LM2 cells following individual knockdown of GATA6 and TGFB2-AS1, as well as simultaneous knockdown of both genes, and corresponding quantitative analysis (right) of these soft agar colony formation results. (**G**) The growth curves of LM2 cells following individual knockdown of GATA6 and TGFB2-AS1, as well as simultaneous knockdown of both genes

To investigate whether GATA6 regulates TNBC aggressiveness via repression of TGFB2-AS1, we first employed LM2 and SUM159PT cells, which exhibit relatively high basal GATA6 and low TGFB2-AS1 expression. were used. Transfection with two distinct siRNAs targeting GATA6 significantly upregulated TGFB2-AS1 expression and markedly downregulated its downstream target genes TGFβ2 and SOX2—both of which are negatively regulated by TGFB2-AS1—at both mRNA and protein levels in LM2 and SUM159PT cells (Fig. 3A-B). Consistently, siGATA6 transfection markedly suppressed cellular migration, invasion, plate colony formation, soft agar colony formation, and proliferation in both cell lines (Supplementary Fig. 3A-J), in agreement with previous findings [12]. To determine whether GATA6 influences the subcellular distribution of TGFB2-AS1, we performed nuclear-cytoplasmic fractionation followed by qPCR in GATA6-knockdown LM2 and GATA6-overexpressing MDA-231 cells. GATA6 depletion increased TGFB2-AS1 levels in both nuclear and cytoplasmic, whereas GATA6 overexpression reduced TGFB2-AS1 in both compartments (Supplementary Fig. 4A). Next, we assessed the functional impact of this regulation on chromatin occupancy performing ChIP assays using an anti-SMARCA4 antibody. GATA6 knockdown significantly reduced SMARCA4 binging to the promoters of TGFβ2 and SOX2 compared to control cells (Fig. 3C). consistent with our prior finding that nuclear TGFB2-AS1 directly interferes with SMARCA4 recruitment to theses loci [20].

To conform that GATA6 exerts its oncogenic effects via TGFB2-AS1, we performed co-knockdown of GATA6 and TGFB2-AS1 in LM2 cells. Notably, simultaneous silencing of TGFB2-AS1 significantly reversed the siGATA6-induced suppression of TGFβ2 and SOX2 at both mRNA and protein levels (Supplementary Fig. 4B–C, quantified band gray values; Fig. 3D). Accordingly, the inhibitory effects of siGATA6 on migration (Supplementary Fig.  4 D), invasion (Fig. 3E), soft agar colony formation (Fig. 3F), plate colony formation (Supplementary Fig. 4E), and proliferation (Fig. 3G) were substantially rescued upon co-transfection with siTGFB2-AS1. Collectively, these data indicate that GATA6 transcriptionally represses TGFB2-AS1, thereby relieving its inhibition on SMARCA4 and enabling activation of TGFβ2 and SOX2, which collectively drive malignant phenotypes in TNBC cells.

### Overexpression of GATA6 promote TNBC malignancy through TGFB2-AS1

**Fig. 4:**
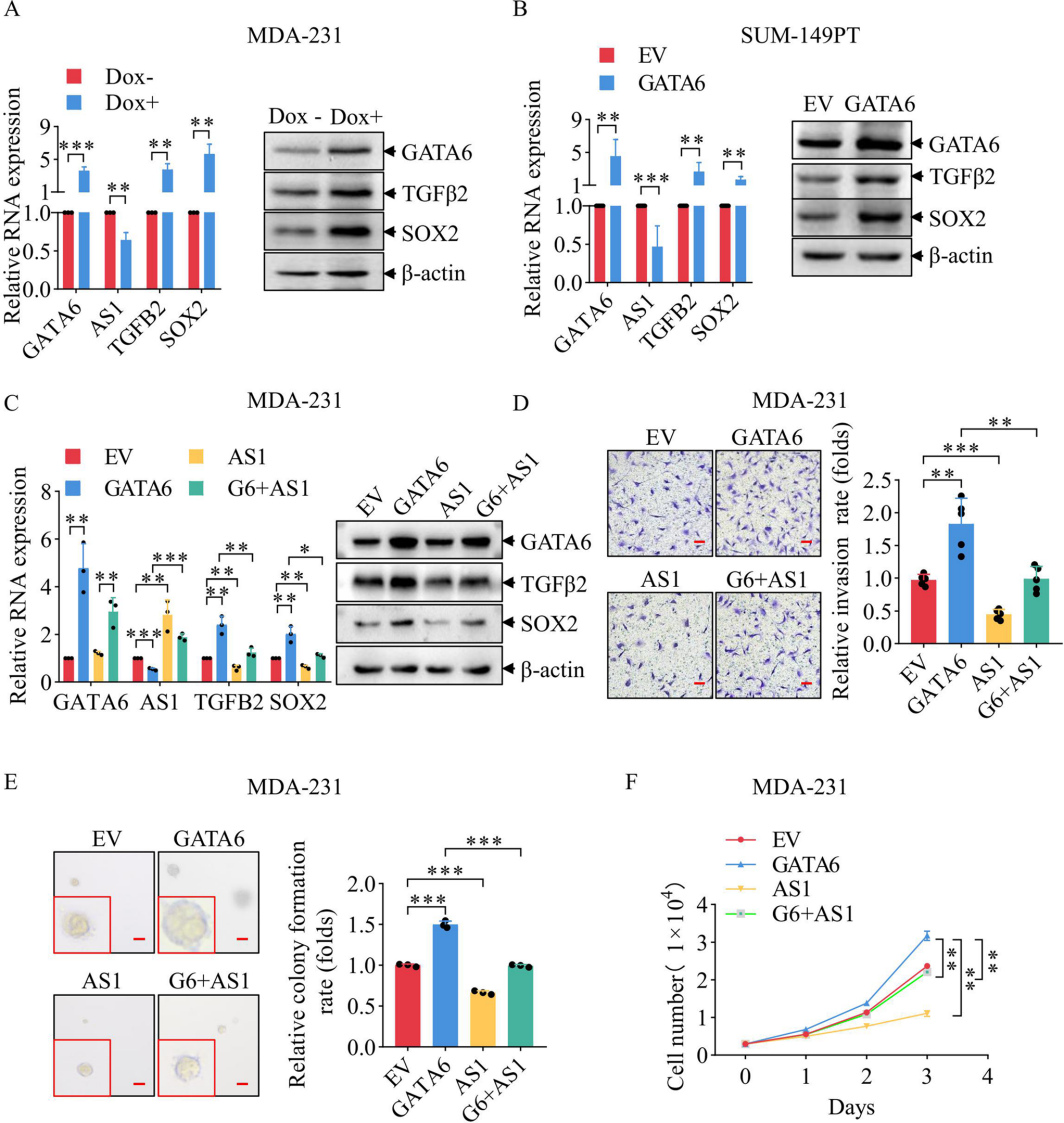
Overexpression of GATA6 promote TNBC malignancy through TGFB2-AS1. (**A**) The RNA (left) and protein (right) expression levels of GATA6, TGFB2-AS1, TGFβ2, and SOX2 after Dox-induced exogenous GATA6 in MDA-231 cells with β-actin as the internal reference. (**B**) The RNA (left) and protein (right) expression levels of GATA6, TGFB2-AS1, TGFβ2, and SOX2 in SUM149PT cells after GATA6 overexpression with β-actin as the internal reference. (**C**) The RNA (left) and protein (right) expression levels of GATA6, TGFB2-AS1, TGFβ2, and SOX2 of MDA-231 cells following individual overexpression of GATA6 and TGFB2-AS1, as well as simultaneous overexpression of both genes with β-actin as the internal reference. (**D**) Illustrative photographs (left, scale: 50 μm) showing the invasion capacity of MDA-231 cells following individual overexpression of GATA6 and TGFB2-AS1, as well as simultaneous overexpression of both genes, and corresponding statistical analysis (right) of these invasion assays. (**E**) Representative images (left, scale: 50 μm) showing the soft agar colony formation capacity of MDA-231 cells following individual overexpression of GATA6 and TGFB2-AS1, as well as simultaneous overexpression of both genes, and corresponding quantitative analysis (right) of these soft agar colony formation results. (**F**) The growth curves of MDA-231 cells following individual overexpression of GATA6 and TGFB2-AS1, as well as simultaneous overexpression of both genes

To further confirm that GATA6 promotes TNBC malignancy through TGFB2-AS1, we used doxycycline (Dox) inducible GATA6-overexpressing MDA-231cells (Supplementary Fig. 5) and GATA6-overexpressing SUM149PT cells. Upon exogenous overexpression of GATA6, TGFB2-AS1 RNA levels were significantly reduced, accompanied by marked increases in both mRNA and protein levels of TGFβ2 and SOX2 compared to controls (Fig. 4A and B). Functional assays revealed that GATA6 overexpression significantly enhanced cell migration (Supplementary Fig. 6A, F), invasion (Supplementary Fig. 6B, G), colony formation on plates (Supplementary Fig. 6C, H) and in soft agar (Supplementary Fig. 6D, I), as well as proliferation (Supplementary Fig. 6E, J) in both MDA-231 and SUM149PT cells.

Next, we co-expressed GATA6 and TGFB2-AS1 exogenously in MDA-231 cells. The GATA6-induced upregulation of TGFβ2 and SOX2 expression was effectively rescued at both mRNA and protein levels upon TGFB2-AS1 restoration (Fig. 4C). Consistently, the enhanced migratory capacity (Supplementary Fig. 6K), invasive potential (Fig. 4D), colony formation on plates (Supplementary Fig. 6L) and in soft agar (Fig. 4E), and proliferative ability (Fig. 4F) driven by GATA6 were significantly attenuated upon TGFB2-AS1 restoration. These findings collectively demonstrate that GATA6 promotes TNBC progression by suppressing TGFB2-AS1 expression.

### TGFB2-AS1 antagonizes GATA6-driven TNBC metastasis in vivo

**Fig. 5:**
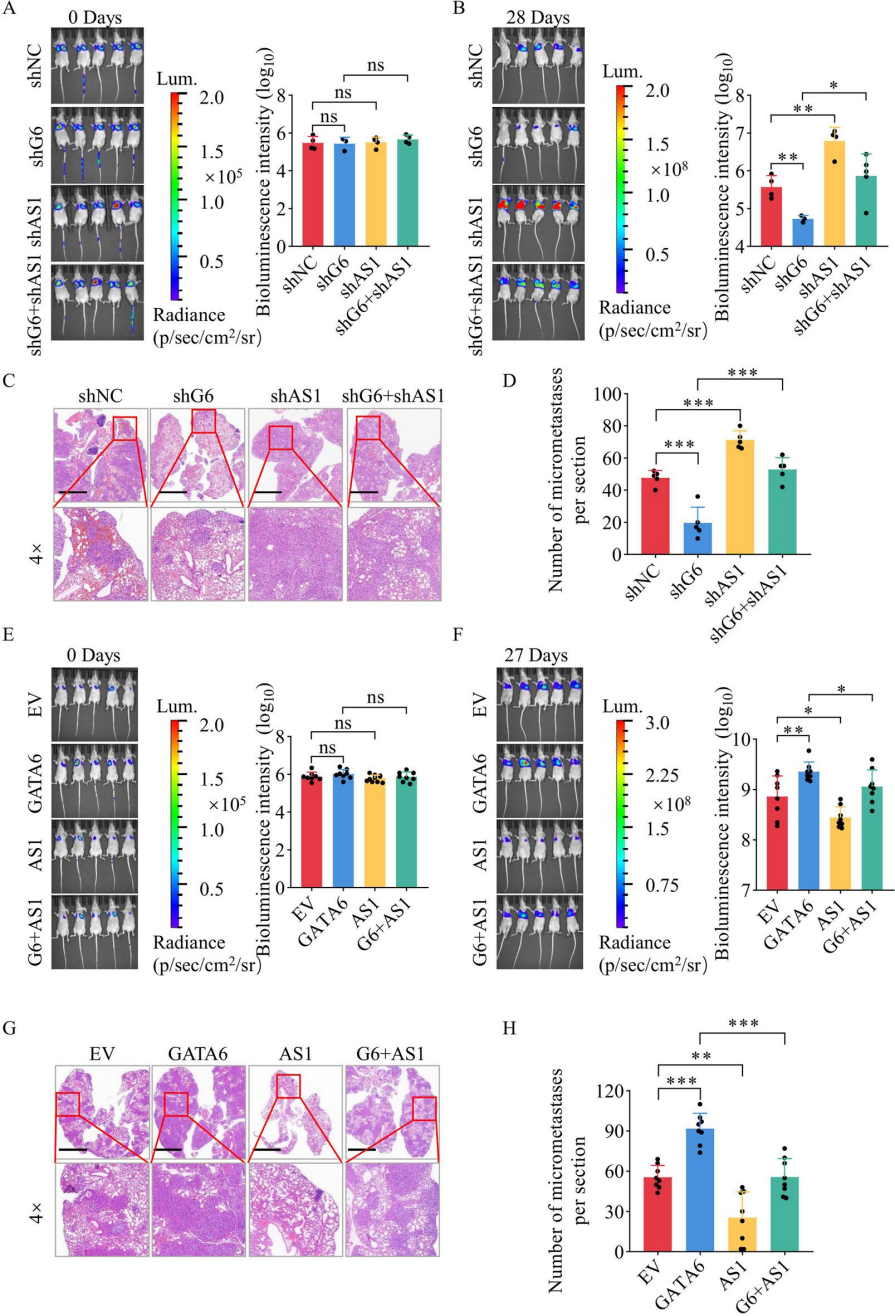
TGFB2-AS1 antagonizes GATA6-driven TNBC metastasis in vivo. (**A**) Representative bioluminescence images (left) of mice injected via the tail vein with different LM2 cell lines (LM2-shNC, LM2-shG6, LM2-shAS1, LM2-shG6 + shAS1) on day 0, and corresponding statistical analysis of bioluminescence intensity in the mouse lungs (right). (**B**) Representative bioluminescence images (left) captured on day 28, showing mice injected with the specified LM2 cell lines, and statistical summary of the bioluminescence intensity observed in the lungs of these mice (right). The experiment was repeated twice. (**C**) Representative images of mouse lung tissues stained with hematoxylin and eosin (scale: 2.5 mm) on day 28 after tail vein injection with different LM2 cell lines. (**D**) Statistical summary of the micrometastatic foci in the lungs of these mice. (**E**) Representative bioluminescence images (left) of mice injected via the tail vein with different LM2 cell lines (LM2-EV, LM2-G6, LM2-AS1, LM2-G6 + AS1) on day 0, and corresponding statistical analysis of bioluminescence intensity in the mouse lungs (right). (**F**) Representative bioluminescence images (left) captured on day 27, showing mice injected with the specified LM2 cell lines, and statistical summary of the bioluminescence intensity observed in the lungs of these mice (right). (**G**) Representative images of mouse lung tissues stained with hematoxylin and eosin (scale: 2.5 mm) on day 27 after tail vein injection with different LM2 cell lines. (**H**) Statistical summary of the micrometastatic foci in the lungs of these mice

To delineate the role of TGFB2-AS1 in GATA6-driven TNBC metastasis in vivo, we generated stable LM2 cell lines with knockdown GATA6 (LM2-shG6), TGFB2-AS1 (LM2-shAS1), or both (LM2-shG6 + shAS1). Following tail vein injection, lung metastasis was monitored by bioluminescence imaging (BLI). Comparable BLI signal intensities at day 0 (*n* = 5 per group), were confirmed equivalent initial lung seeding (Fig. 5A). Weekly BLI from week 2 onward revealed that LM2-shAS1 cells exhibited enhanced metastatic progression, whereas LM2-shG6 cells showed suppressed metastatic progression to controls. Notably, concurrent knockdown of TGFB2-AS1 in LM2-shG6 group reversed the metastasis-suppressive phenotype induced by GATA6 silencing (Supplementary Fig. 7A).

On day 28, mice were euthanized following the final BLI scan. Lungs were harvested, weighed, and imaged. The lung-to-body weight ratio (Supplementary Fig. 7B) and representative macroscopic images of lung tissues (Supplementary Fig. 7C) demonstrated significantly increased tumor burden in LM2-shAS1 group and reduced burden in LM2-shG6 group. Concurrent TGFB2-AS1 knockdown in LM2-shG6 + shAS1 group reversed the anti-metastatic effect of GATA6 silenced (Fig. 5B), a finding corroborated by H&E staining (Fig. 5C) and lesion quantification (Fig. 5D), confirming that shTGFB2-AS1 reverses shGATA6-leaded metastasis suppression.

Reciprocally, LM2 cells overexpressing GATA6 (LM2-G6), TGFB2-AS1 (LM2-AS1), or both (LM2-G6 + AS1) were injected (*n* = 8 per group). Day 0 BLI confirmed uniform seeding (Fig. 5E). By day 27, LM2-G6 mice developed extensive metastases, while LM2-AS1 mice showed attenuated disease. Notably, co-expression of TGFB2-AS1 in LM2-G6 + AS1 mice markedly reduced metastatic burden—evident in BLI (Supplementary Fig. 7D, Fig. 5F), the lung-to-body weight ratio (Supplementary Fig. 7E), macroscopic appearance (Supplementary Fig. 7F), and histopathology (Fig. 5G-H)—restoring a phenotype comparable to controls. Collectively, these data establish TGFB2-AS1 as a critical effector of GATA6-dependent TNBC progression in vivo.

## Discussion

TNBC continues to be a major research priority given its limited treatment modalities and elevated rates of recurrence and metastasis. Our previous work demonstrated that TGFB2-AS1 interacts with SMARCA4, a core subunit of the SWI/SNF chromatin remodeling complex, to inhibit TGFβ2 and SOX2 expression, thereby attenuating TNBC malignancy. While TGFB2-AS1 downregulation correlates with aggressive TNBC phenotypes and poor patient prognosis, the mechanisms underlying its suppression remain undefined.

Through transcription factor prediction algorithms and TNBC prognostic correlation analyses, we identified GATA6, YY1, and SP1 as candidate regulators of TGFB2-AS1. TCGA data analysis revealed that among these factors, only GATA6 expression significantly correlated with adverse clinical outcomes in breast cancer patients (Supplementary Fig. 1B-D). Cellular-level investigations confirmed an inverse relationship between GATA6 and TGFB2-AS1 expression levels. Mechanistically, GATA6 bound to three distinct sites in the TGFB2-AS1 promoter, effectively repressing its transcription and consequently upregulating TGFβ2 and SOX2. Functional assays demonstrated that TGFB2-AS1 mediates GATA6-driven oncogenic phenotypes in TNBC cells, including enhanced proliferation, migration, invasion, and clonogenicity. Importantly, in vivo studies using a breast cancer lung metastasis mouse model established TGFB2-AS1’s role in counteracting GATA6-mediated metastatic dissemination.

As a member of the evolutionarily conserved zinc finger transcription factor family, GATA6 recognizes the (G/A)GATA(A/T) motif to regulate endodermal lineage specification and epithelial maintenance during embryogenesis. While GATA6 typically preserves epithelial characteristics by suppressing EMT in malignancies such as pancreatic and lung cancers, our findings reveal a context-dependent pro-metastatic role for GATA6 in TNBC through repression of TGFB2-AS1. Clinically, the GATA6 high/TGFB2-AS1-low expression signature emerged as a robust prognostic indicator for TNBC patients.

To further evaluate the clinical relevance of this signature, we performed multivariate Cox regression analysis adjusting for key clinical covariates, including tumor stage and breast disease history. High GATA6 and low TGFB2-AS1 expression showed a non-significant trended toward worse overall survival (HR = 1.42, 95% CI: 0.92–2.18; *p* = 0.11), consistent with our univariate Kaplan–Meier results—neither marker reached statistical significance as an independent predictor after adjustment. This suggests that the prognostic signal of the GATA6/TGFB2-AS1 axis may be partially confounded by or operate in concert with established clinicopathological factors, particularly tumor stage. Nevertheless, the biological consistency across in vitro, in vivo, and patient-derived data underscores the functional importance of this regulatory axis in TNBC progression.

In conclusion, this study elucidates a tumor-promoting mechanism of GATA6 via transcriptional suppression of TGFB2-AS1 in TNBC. These results not only extend our previous findings but also identify the GATA6-high/TGFB2-AS1-low expression signature as a clinically associated and biologically meaningful pattern in TNBC patients, which may inform risk stratification and guide future therapeutic strategies.

## Supplementary Information

Below is the link to the electronic supplementary material.

Supplementary Material 1

Supplementary Material 2

Supplementary Material 3

Supplementary Material 4

Supplementary Material 5

Supplementary Material 6

Supplementary Material 7

## Data Availability

No datasets were generated or analysed during the current study.
