# Supplementary material for: Transcriptional repression of TGFB2-AS1 by GATA6 drives triple-negative breast cancer metastasis
Source: Cell Oncol (Dordr). 2026 Apr 1;49(2):64. doi: 10.1007/s13402-026-01195-5 (PMC13043960; doi:10.1007/s13402-026-01195-5)
Supplement: Supplementary file 5 — Supplementary Material 5 [file 13402_2026_1195_MOESM5_ESM.docx]

**Supplementary table 5: Correlation of GATA6 with Clinical Characteristics: An Analysis of Breast Cancer Patients from Ruijin Hospital**

| Clinical Feature | GATA6（high） | GATA6(low) | χ² | P-value |
| --- | --- | --- | --- | --- |
| Menopausal Status |  |  | 3.956 | 0.0609^a^ |
| Premenopausal | 29 | 18 |  |  |
| Postmenopausal | 31 | 41 |  |  |
| Histological Grade |  |  | 0.651 | 0.420^a^ |
| I | 14 | 15 |  |  |
| II-III | 40 | 30 |  |  |
| TNM Stage |  |  | 4.15 | 0.042^a^ |
| Stage I | 14 | 25 |  |  |
| Stage II-III | 42 | 33 |  |  |
| History of Breast Disease |  |  | 0.0309 | 0.86^a^ |
| Yes | 12 | 13 |  |  |
| No | 47 | 47 |  |  |
| Age |  |  | 2.791 | 0.095^a^ |
| ＜50 | 25 | 16 |  |  |
| ≥50 | 35 | 43 |  |  |
| Lymph Node Metastasis |  |  | 0.0773 | 0.781^a^ |
| Positive | 19 | 17 |  |  |
| Negative | 41 | 41 |  |  |

^a^Pearson χ²
