# Supplementary material for: Transcriptional repression of TGFB2-AS1 by GATA6 drives triple-negative breast cancer metastasis
Source: Cell Oncol (Dordr). 2026 Apr 1;49(2):64. doi: 10.1007/s13402-026-01195-5 (PMC13043960; doi:10.1007/s13402-026-01195-5)
Supplement: Supplementary file 7 — Supplementary Material 7 [file 13402_2026_1195_MOESM7_ESM.zip › Supplementary figure 3-6.docx]

**Transcriptional repression of TGFB2-AS1 by GATA6 drives triple-negative breast cancer metastasis**

Chang Liu^1, ‡^, Qianru Yu^1, ‡^, Difei Wang^1^, Zheng Duan^1^, Xin Zhang^1^, Jiao Wang^1^, Xiaoyu Qi^1^, Jiayin Ye^1^, Qian Zhao^1^, Jianrong He^2,*^, Cixiang Zhou^1,*^

^‡^These authors contributed equally to this work

Authors’ affiliations: ^1^ Institute for Translational Medicine on Cell Fate and Disease, Shanghai Ninth People' s Hospital, Key Laboratory of Cell Differentiation and Apoptosis of National Ministry of Education, Department of Pathophysiology, Shanghai Jiao Tong University School of Medicine, Shanghai, China; ^2^Department of General Surgery, Comprehensive Breast Health Center, Ruijin Hospital, SJTU-SM, Shanghai 200025, China.

Corresponding author: Jianrong He, Cixiang Zhou; 200025 Shanghai, China; hejrong6636@163.com; zhoucx@shsmu.edu.cn.

**
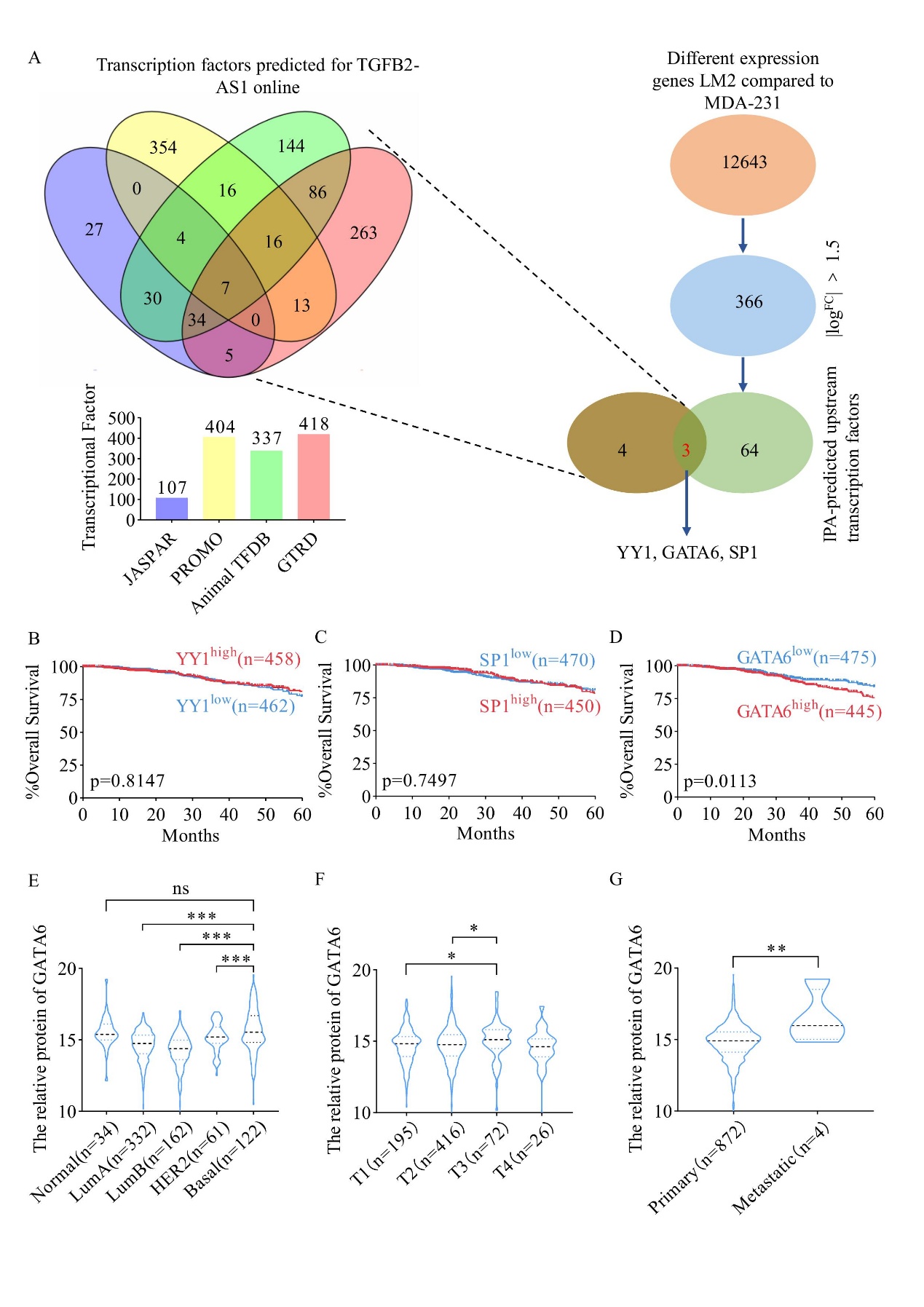
 Supplementary Figure 1** Screening the candidate transcriptional factor that regulates TGFB2-AS1 utilizing bioinformatics. (A) Three transcription factors (YY1, SP1, GATA6) were selected unbiasedly by taking intersections. The intersection was taken between the 7 potential transcription factors identified simultaneously by four prediction software (GTRD，PROMO, JASPAR, Animal TFDB) and the 67 upstream regulatory transcription factors derived from the differential expression profile analysis of MDA-231 and LM2 cells using IPA. (B, C and D) Respective survival analyses of YY1, SP1, and GATA6 in BRCA based on TCGA database. (E) GATA6 protein expression in breast cancer subtypes. (F) Comparison of GATA6 protein expression across different tumor size groups. (G) Association of GATA6 expression with metastasis in TNBC.

**
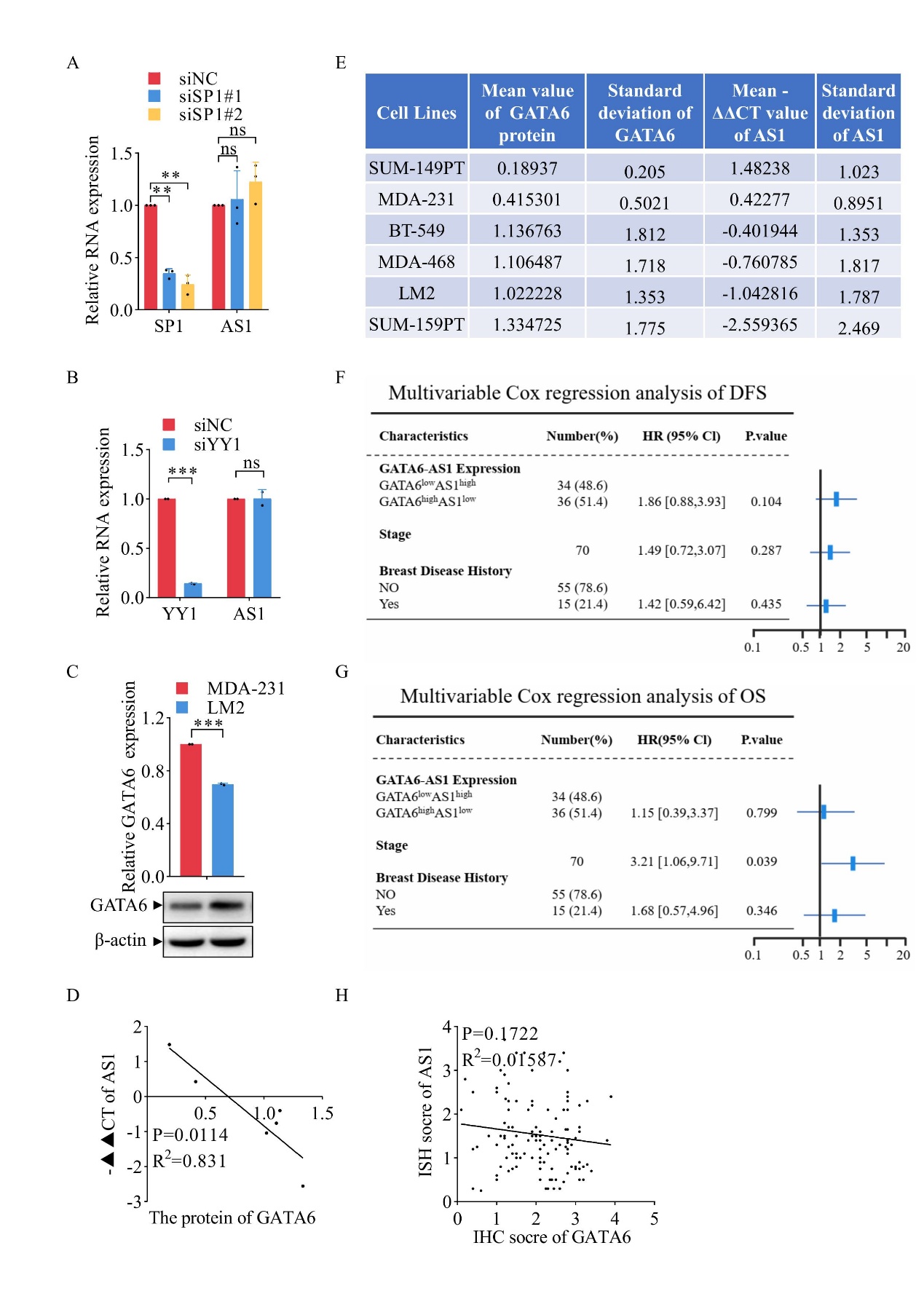
 Supplementary Figure 2** Concurrent high GATA6 and Low TGFB2-AS1 Expression Predicts Aggressive TNBC Phenotype. (A and B) Knockdown of SP1(A) or YY1(B) shows no significant effect on TGFB2-AS1 expression. (C) GATA6 mRNA (up) and protein (down) levels in MDA-231 and LM2 cells. (D) Correlation between GATA6 protein and -▲▲CT of AS1 in seven TNBC cell lines. (E) The expression of GATA6 protein and -▲▲CT of AS1 in seven TNBC cell lines. (F) Multivariable Cox regression analysis of DFS comparing the GATA6^high^/AS1^low^ group to the GATA6^low^/AS1^high^ group. (G) Multivariable Cox regression analysis of OS comparing the GATA6^high^/AS1^low^ group to the GATA6^low^AS1^high^ group. (H) Correlation between GATA6 and AS1 in 119 Ruijin Hospital samples.

**
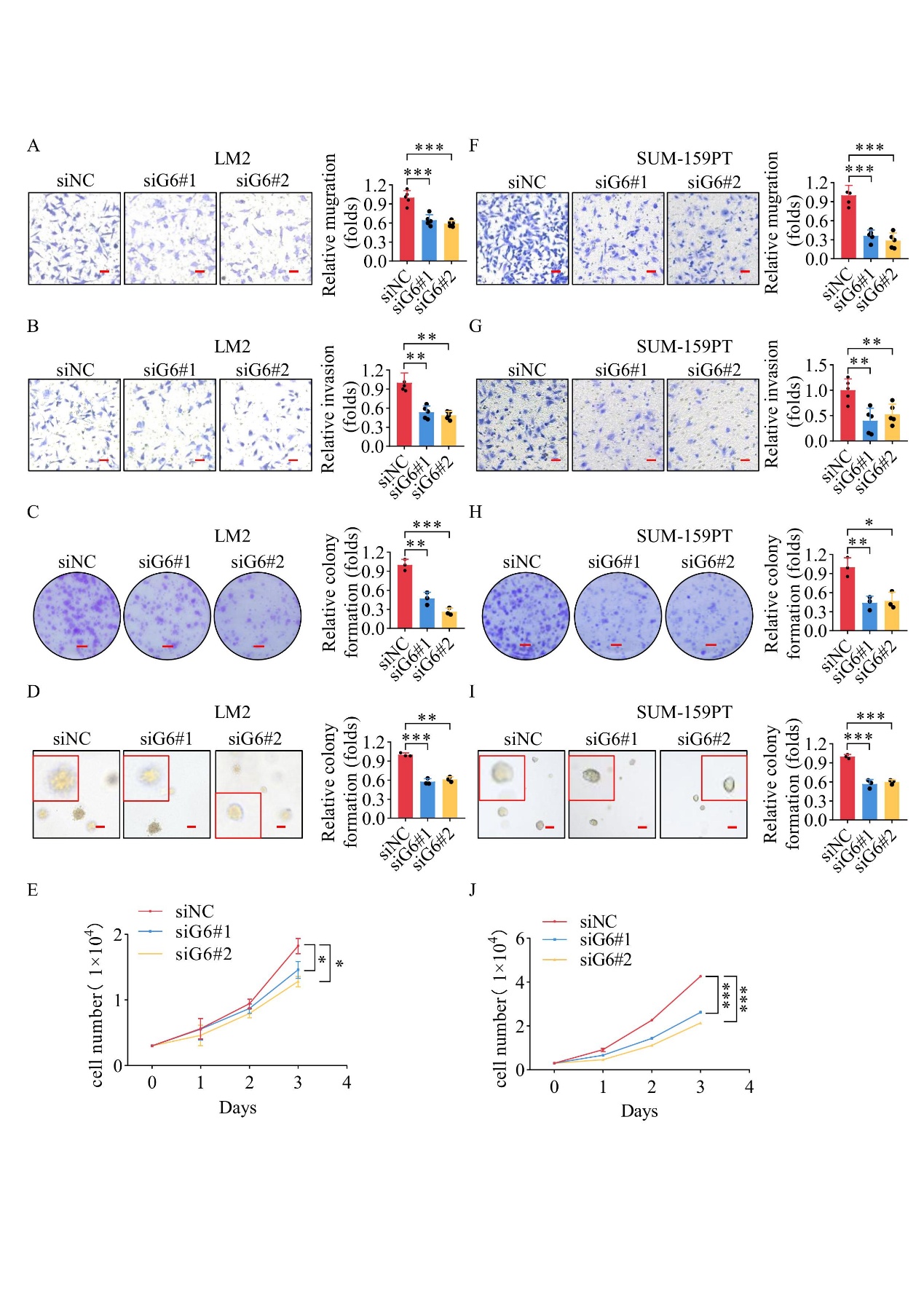
 Supplementary Figure 3** Knockdown of GATA6 suppresses TNBC malignancy. (A and F) Illustrative photographs (left, scale: 50 μm) showing the migration capacity and corresponding quantification in LM2 (A) and SUM‑159PT (F) cells following knockdown of GATA6. (B and G) Illustrative photographs (left, scale: 50 μm) showing the invasion capacity and corresponding quantification in LM2 (B) and SUM‑159PT (G) cells following knockdown of GATA6. (C and H) Illustrative photographs (left, scale: 0.5 cm) showing the plate colony formation capacity and corresponding quantification in LM2 (C) and SUM‑159PT (H) cells following knockdown of GATA6. (D and I) Illustrative photographs (left, scale: 0.5 cm) showing the soft agar colony formation capacity and corresponding quantification in LM2 (D) and SUM‑159PT (I) cells following knockdown of GATA6. (E and J) Growth curves in LM2 (E) and SUM‑159PT (J) cells following knockdown of GATA6.

**
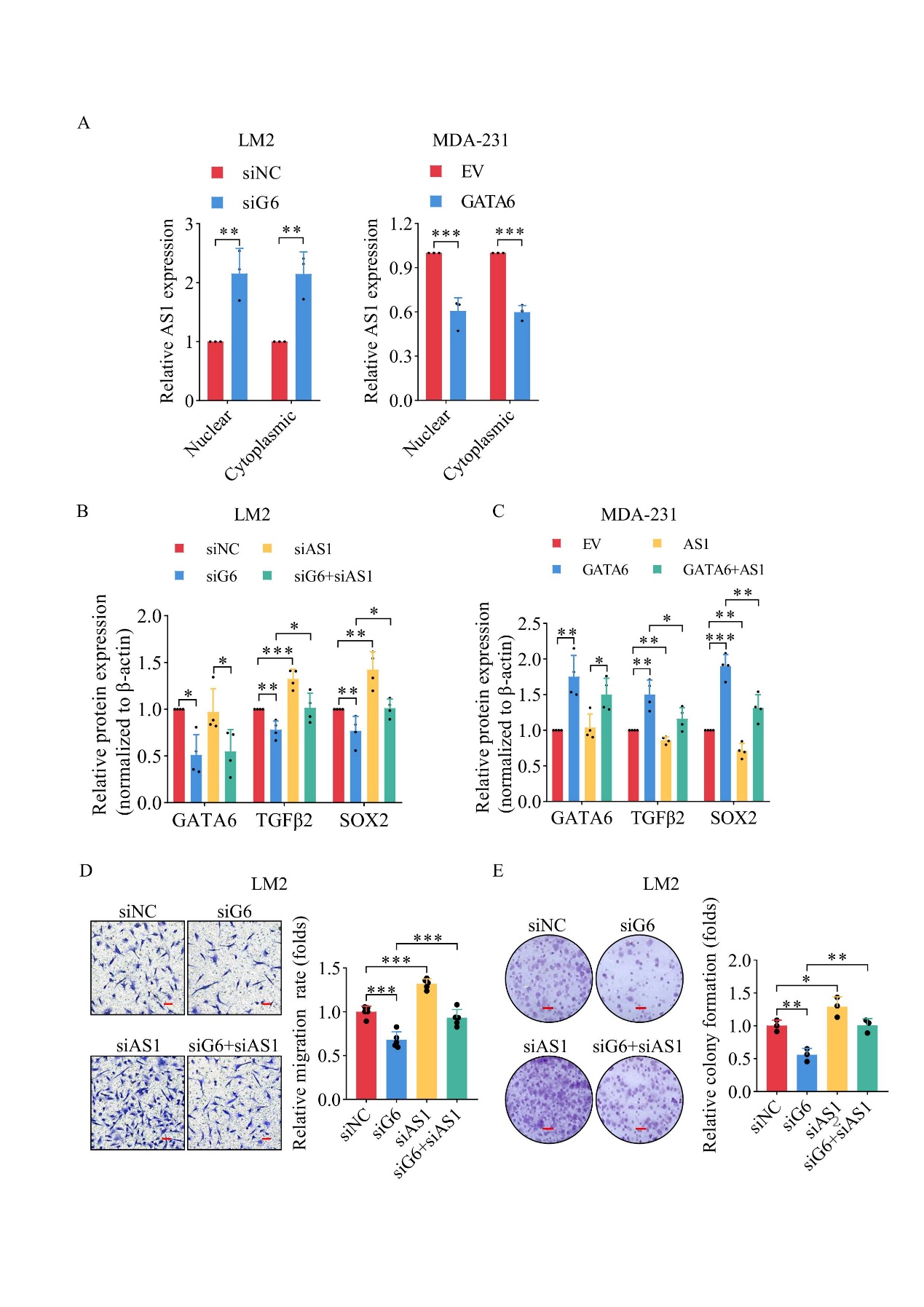
 Supplementary Figure 4** Knockdown of GATA6 suppresses TNBC malignancy through TGFB2-AS1. (A)Relative expression of TGFB2‑AS1 in the nucleus and cytoplasm in LM2 cells following knockdown of GATA6 (left); Relative expression of TGFB2‑AS1 in the nucleus and cytoplasm in MDA-231 cells following overexpression of GATA6 (right). (B)Relative protein expression of GATA6, TGFβ2, and SOX2 in LM2 cells following individual knockdown of GATA6 and TGFB2-AS1, as well as simultaneous knockdown of both genes with β-actin as the internal reference. (C)Relative protein expression of GATA6, TGFβ2, and SOX2 in MDA-231 cells individual overexpression of GATA6 and TGFB2-AS1, as well as simultaneous overexpression of both genes with β-actin as the internal reference. (D) Illustrative photographs (left, scale: 50 μm) showing the migration capacity of LM2 cells following individual knockdown of GATA6 and TGFB2-AS1, as well as simultaneous knockdown of both genes, and corresponding statistical analysis (right) of these migration assays. (E) Representative images (left, scale: 0.5 cm) showing the plate colony formation capacity of LM2 cells following individual knockdown of GATA6 and TGFB2-AS1, as well as simultaneous knockdown of both genes, and corresponding quantitative analysis (right) of these plate colony formation results.

**
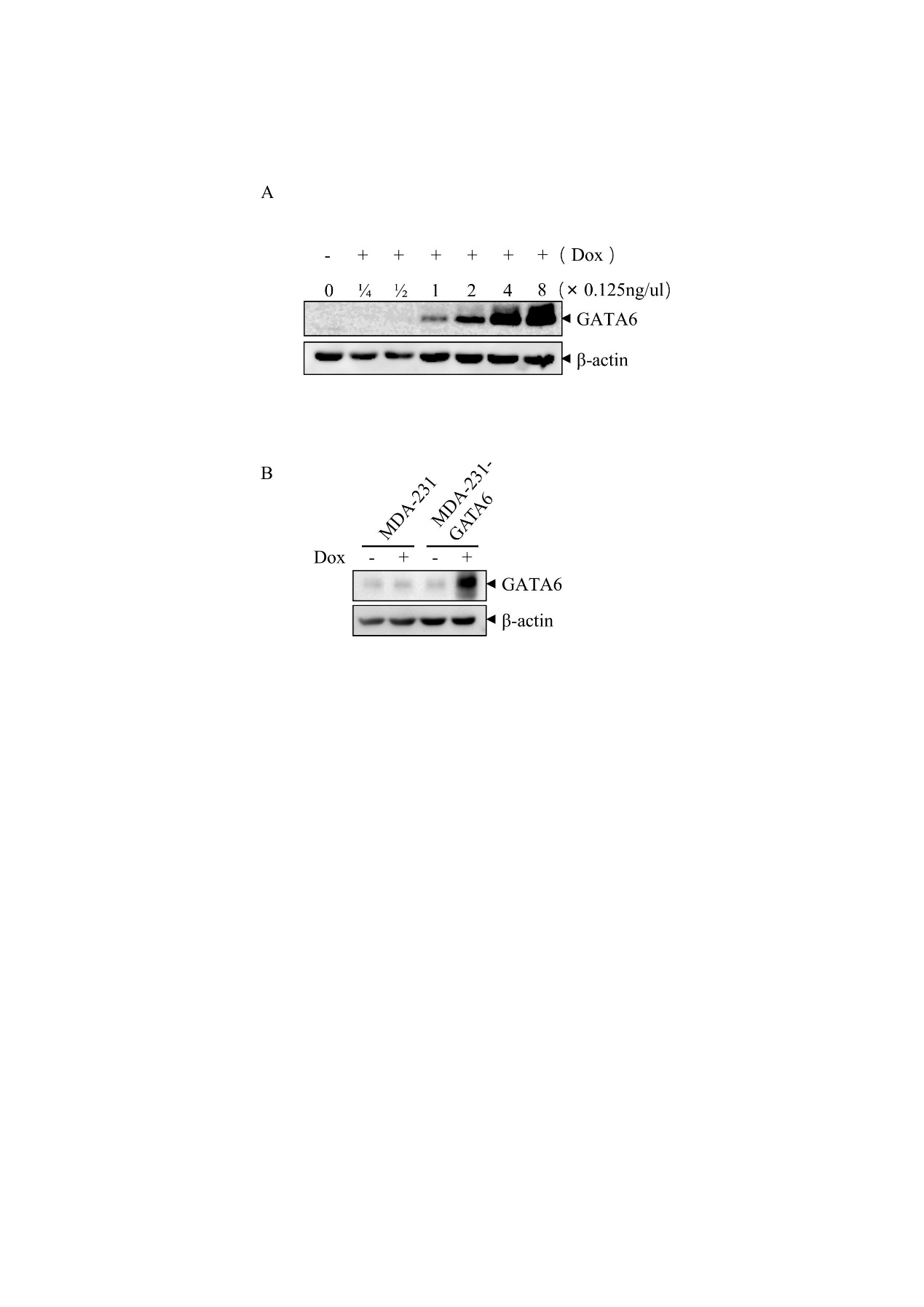
 Supplementary Figure 5** Generated a GATA6-inducible cell line (pINDUCER20-GATA6). (A) GATA6 protein levels 48 h after Dox induction. (B) GATA6 protein levels 48 h in MDA- 231 cells and Dox-induced GATA6 overexpression cells with or without Dox induction for 48 h.

**
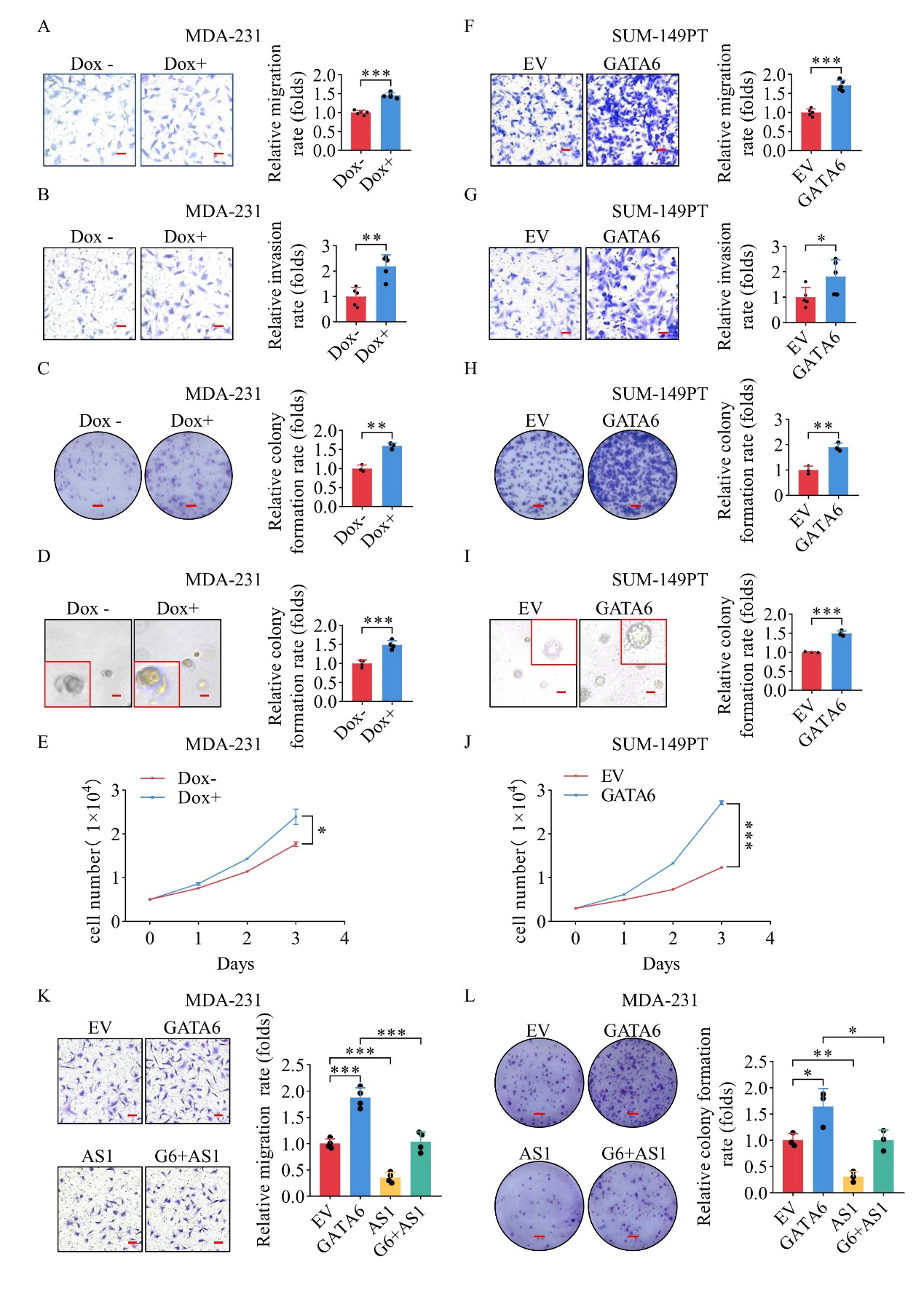
 Supplementary Figure 6** Overexpress of GATA6 promote TNBC malignancy through TGFB2-AS1. (A and F) Illustrative photographs (left, scale: 50 μm) showing the migration capacity and corresponding quantification in MDA-231 (A) and SUM‑149PT (F) cells following overexpression of GATA6. (B and G) Illustrative photographs (left, scale: 50 μm) showing the invasion capacity and corresponding quantification in MDA-231 (B) and SUM‑149PT (G) cells following overexpression of GATA6. (C and H) Illustrative photographs (left, scale: 0.5 cm) showing the plate colony formation capacity and corresponding quantification in MDA-231 (C) and SUM‑149PT (H) cells following overexpression of GATA6. (D and I) Illustrative photographs (left, scale: 0.5 cm) showing the soft agar colony formation capacity and corresponding quantification in MDA-231 (D) and SUM‑149PT (I) cells following overexpression of GATA6. (E and J) Growth curves in MDA-231 (E) and SUM‑149PT (J) cells following overexpression of GATA6. (K) Illustrative photographs (left, scale: 50 μm) showing the migration capacity of MDA-231 cells following individual overexpression of GATA6 and TGFB2-AS1, as well as simultaneous overexpression of both genes, and corresponding statistical analysis (right) of these migration assays. (L) Representative images (left, scale: 0.5 cm) showing the plate colony formation capacity of MDA-231 cells following individual overexpression of GATA6 and TGFB2-AS1, as well as simultaneous overexpression of both genes, and corresponding quantitative analysis (right) of these plate colony formation results.


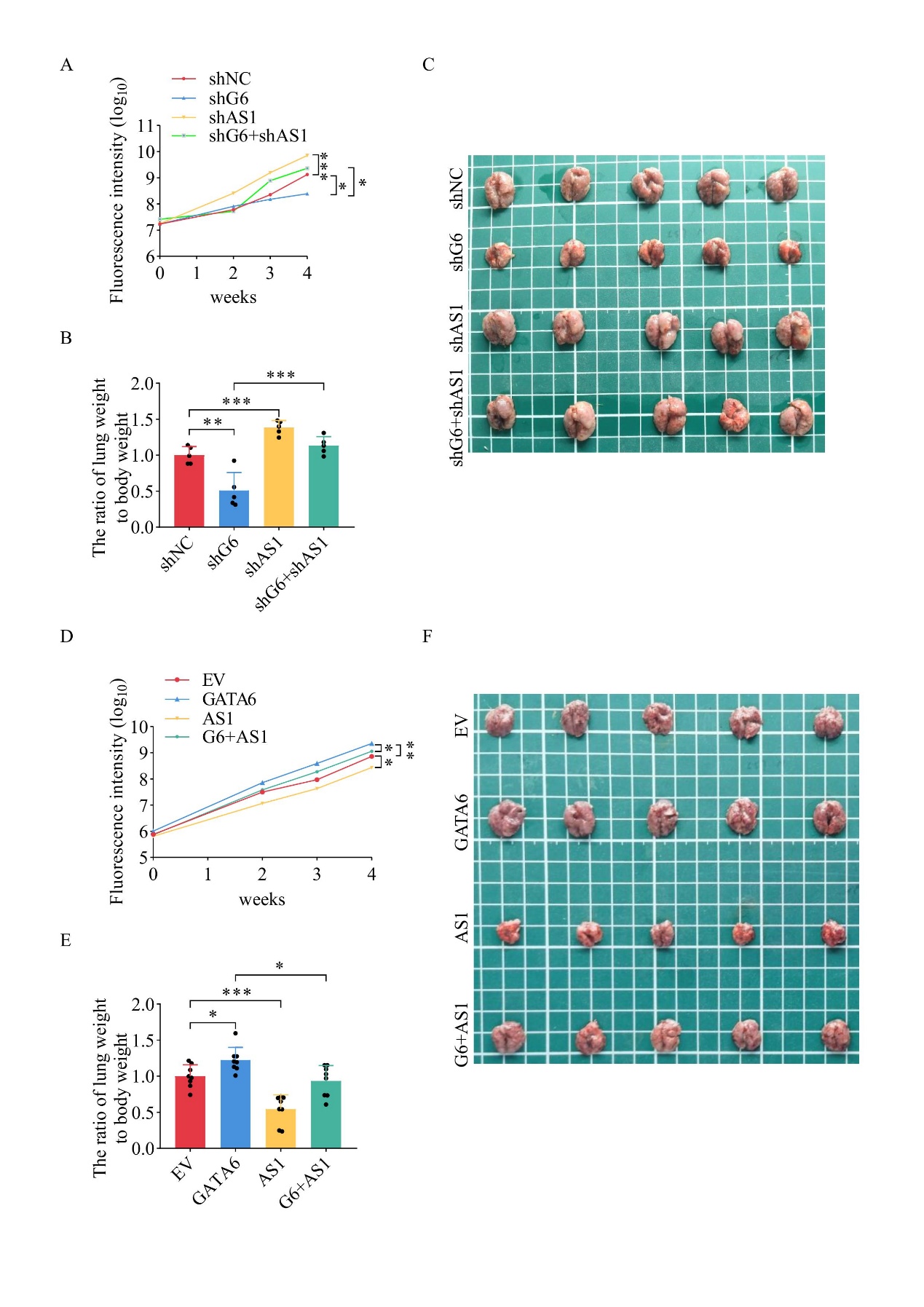


**Supplementary Figure 7** TGFB2-AS1 antagonizes GATA6-driven TNBC metastasis in vivo. (A) Quantitative analysis of weekly lung bioluminescence imaging (BLI) in mice injected with different LM2 cell lines (LM2-shNC, LM2-shG6, LM2-shAS1, LM2-shG6+shAS1). (B) The ratio of lung weight to body weight in mice injected with the specified LM2 cell lines. (C) Excised lung tissues from mice injected via the tail vein with the specified LM2 cell lines on day 28. (D) Quantitative analysis of weekly lung bioluminescence imaging (BLI) in mice injected with different LM2 cell lines (LM2-EV, LM2-G6, LM2-AS1, LM2-G6+AS1). (E) The ratio of lung weight to body weight in mice injected with the specified LM2 cell lines. (F) Excised lung tissues from mice injected via the tail vein with the specified LM2 cell lines on day 27.
