# Supplementary material for: Transcriptional repression of TGFB2-AS1 by GATA6 drives triple-negative breast cancer metastasis
Source: Cell Oncol (Dordr). 2026 Apr 1;49(2):64. doi: 10.1007/s13402-026-01195-5 (PMC13043960; doi:10.1007/s13402-026-01195-5)

Fig. 1A

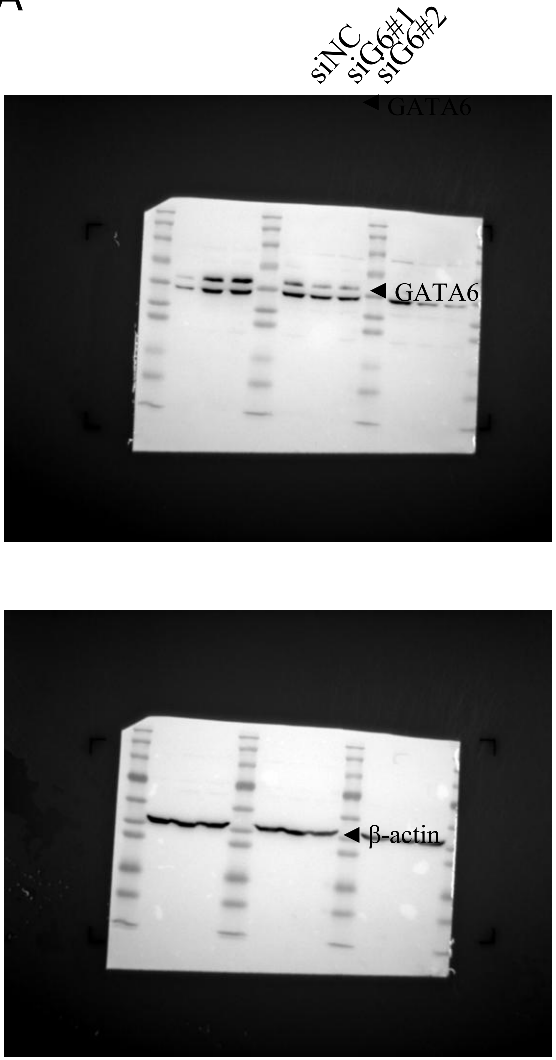

Fig. 1B

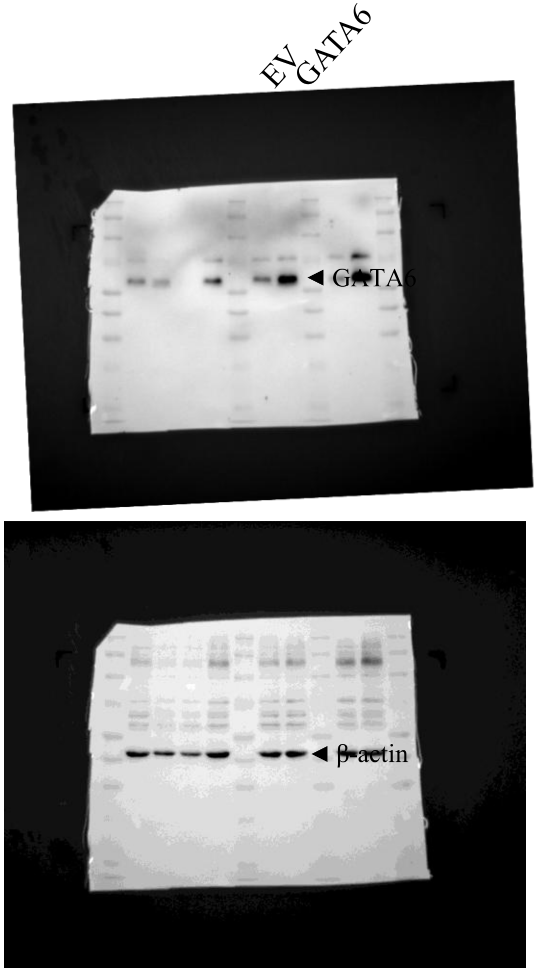

Fig. 1D

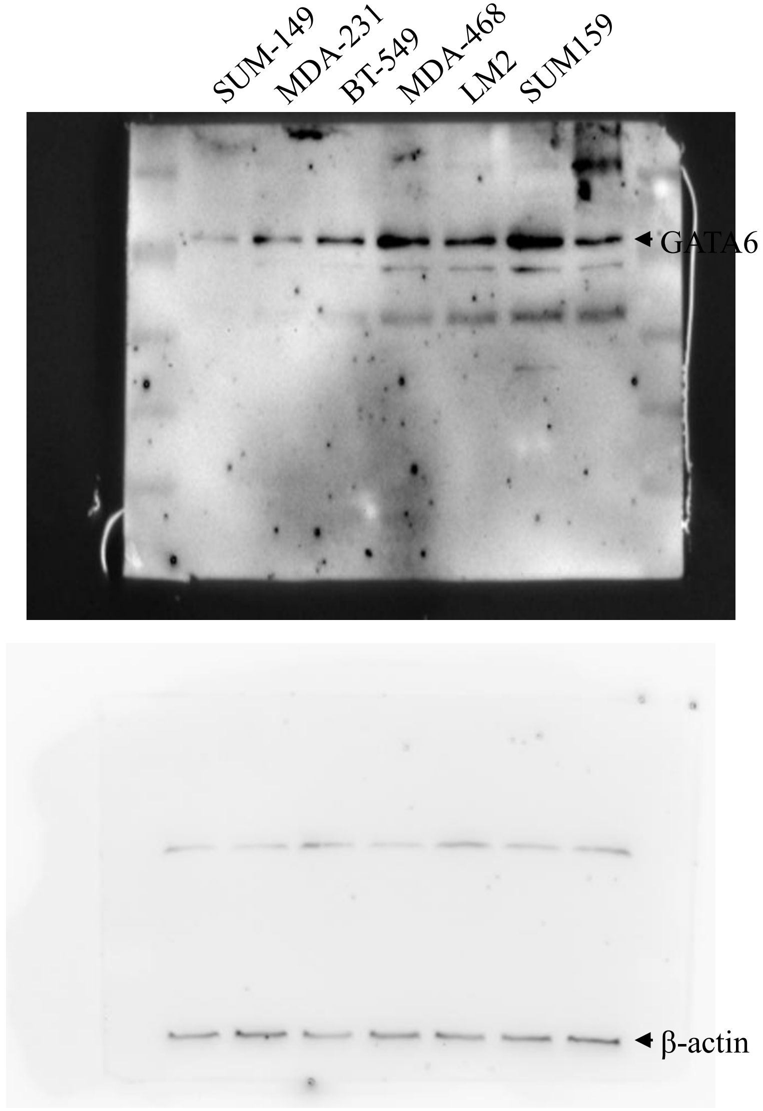
SUM-149
MDA-231
BT-549
MDA-468
LM2
SUM159
GATA6
β-actin

Fig. 1C

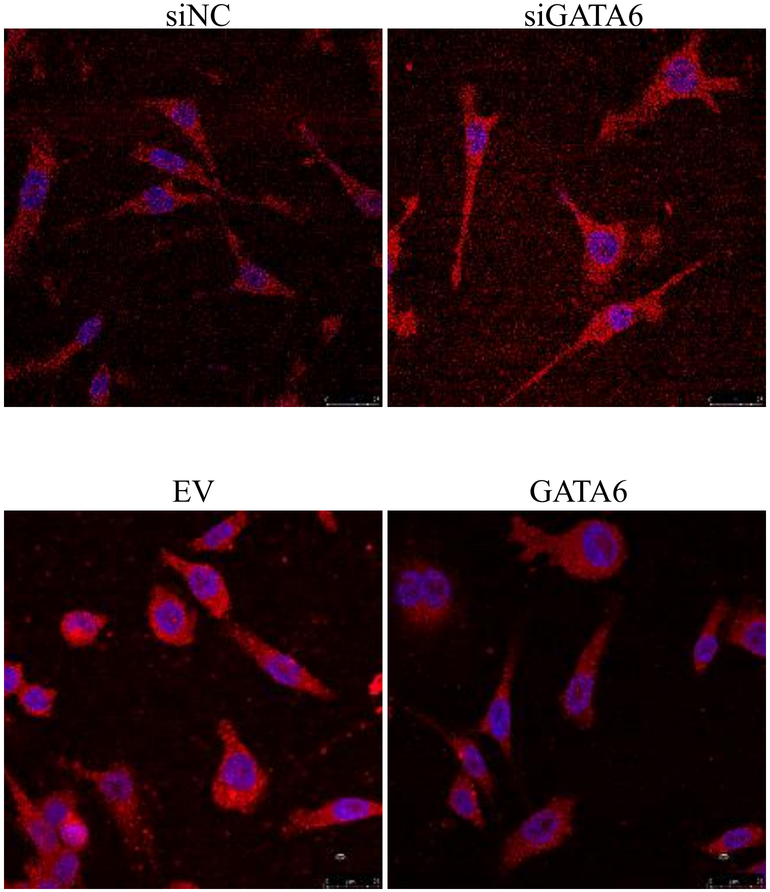
siNC
siGATA6
EV
GATA6

Fig. 2A

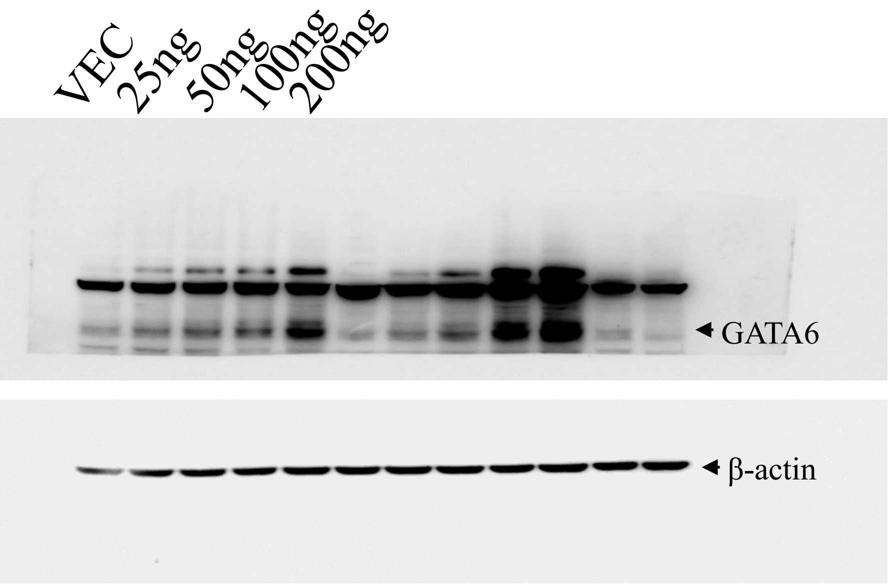

Fig. 2B

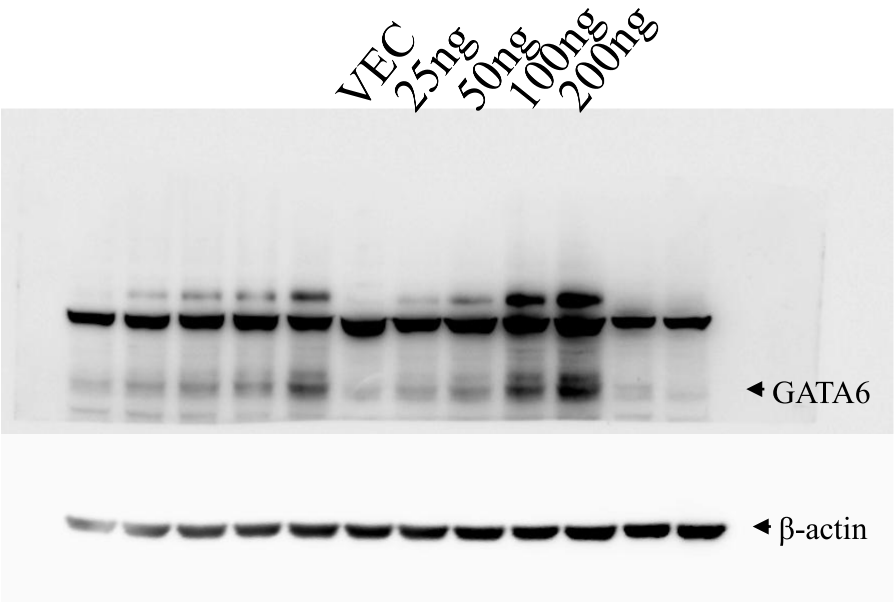

Fig. 2F

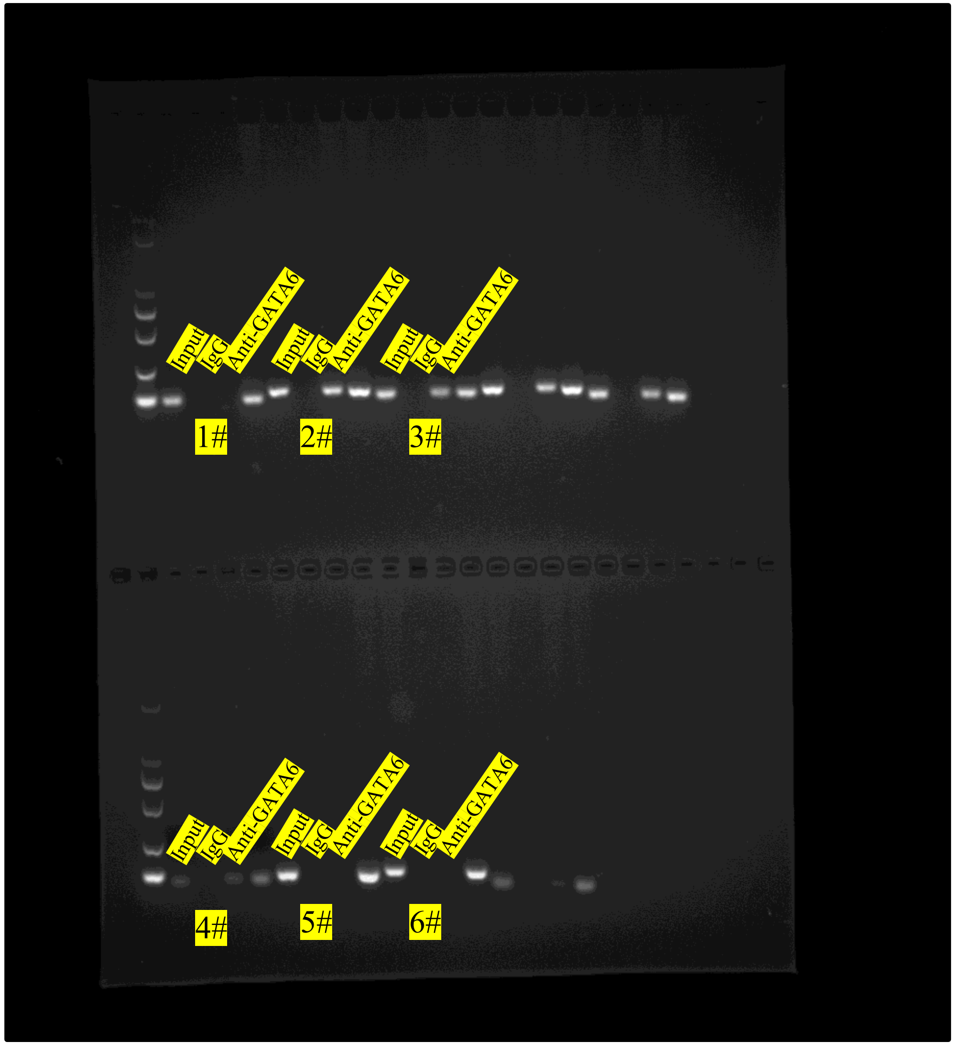

Fig. 3A , Fig. 4A

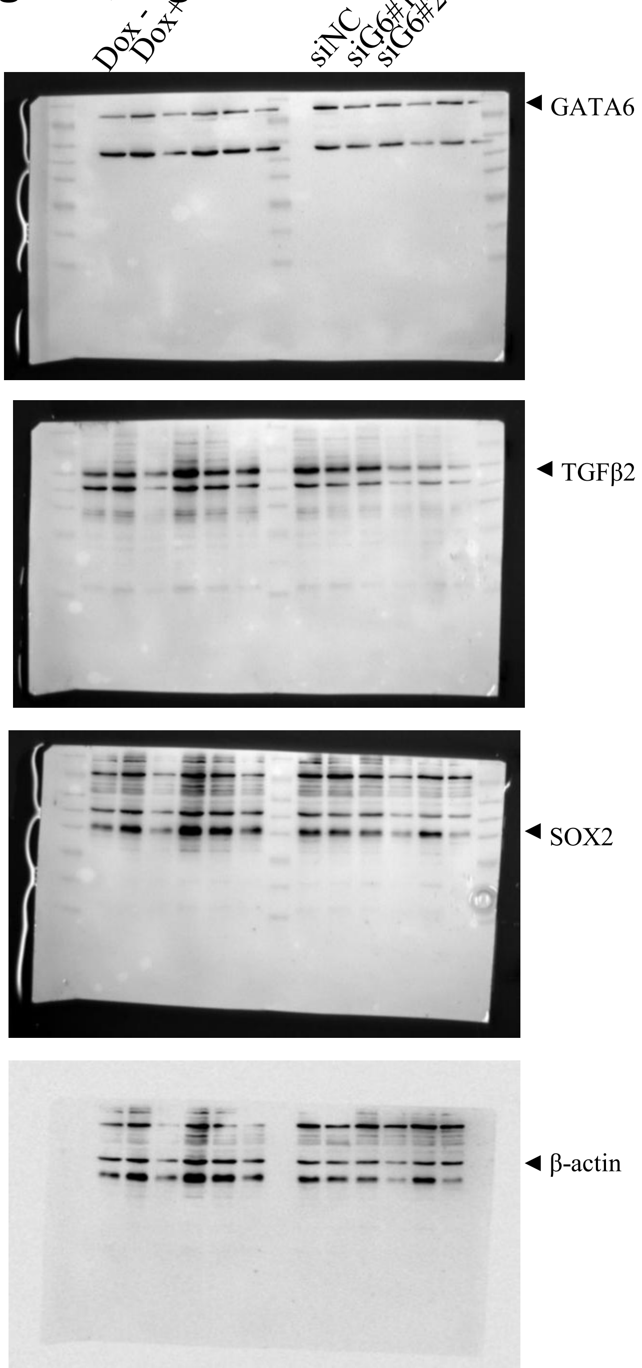

Fig. 3C

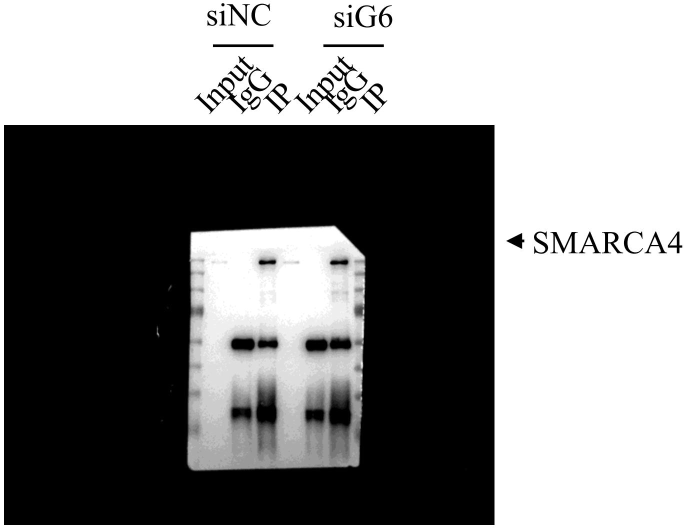

Fig. 3B

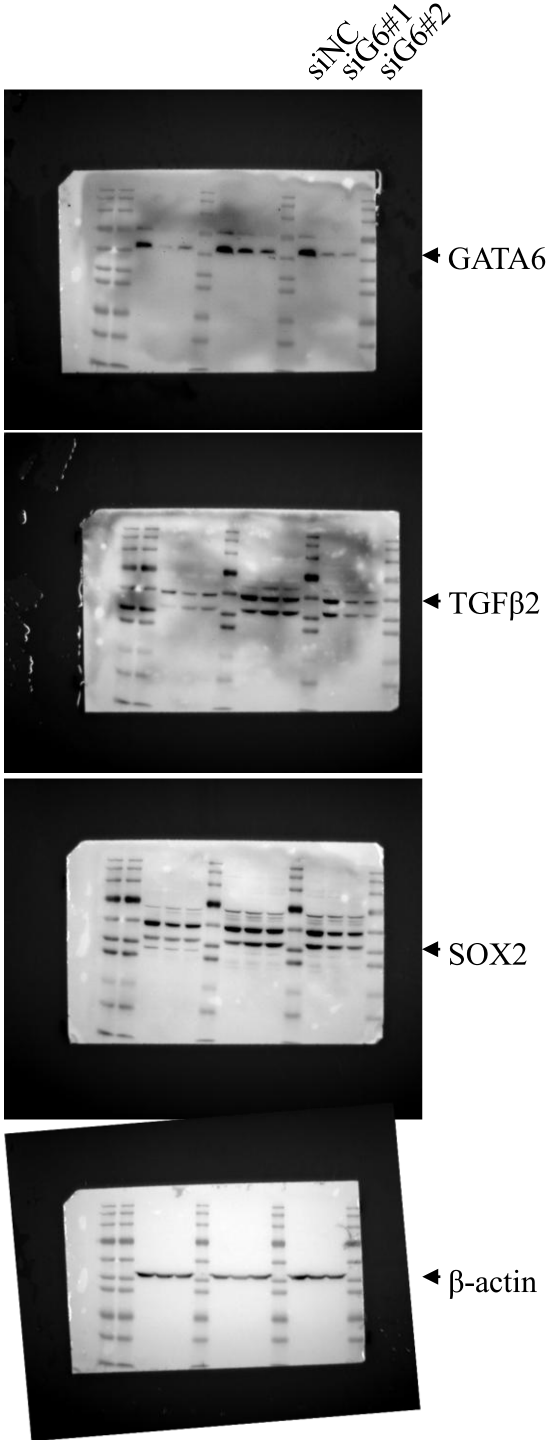

Fig. 3E

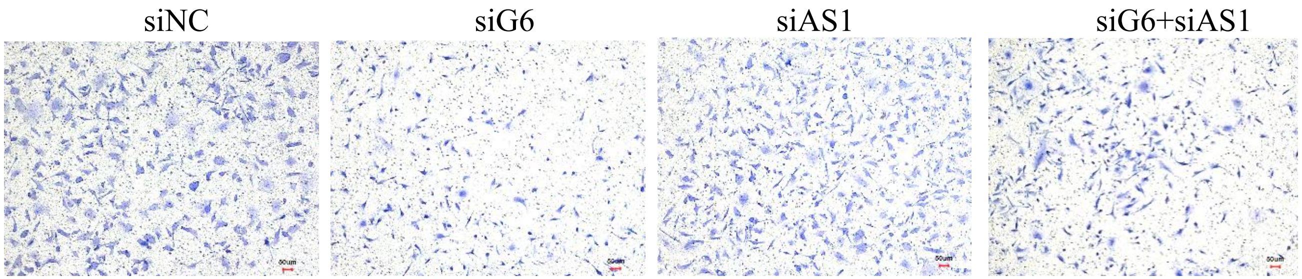

Fig. 3F

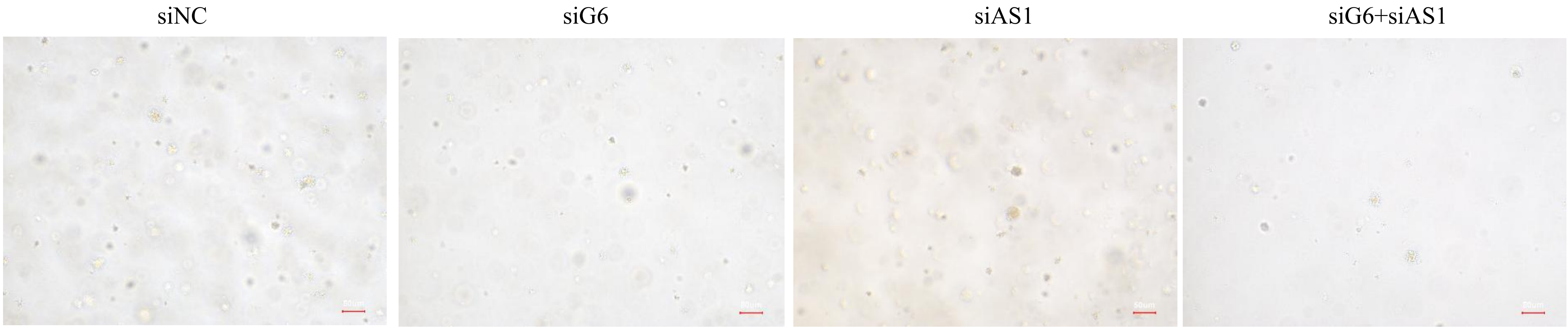

Fig. 3D

Fig. 4B

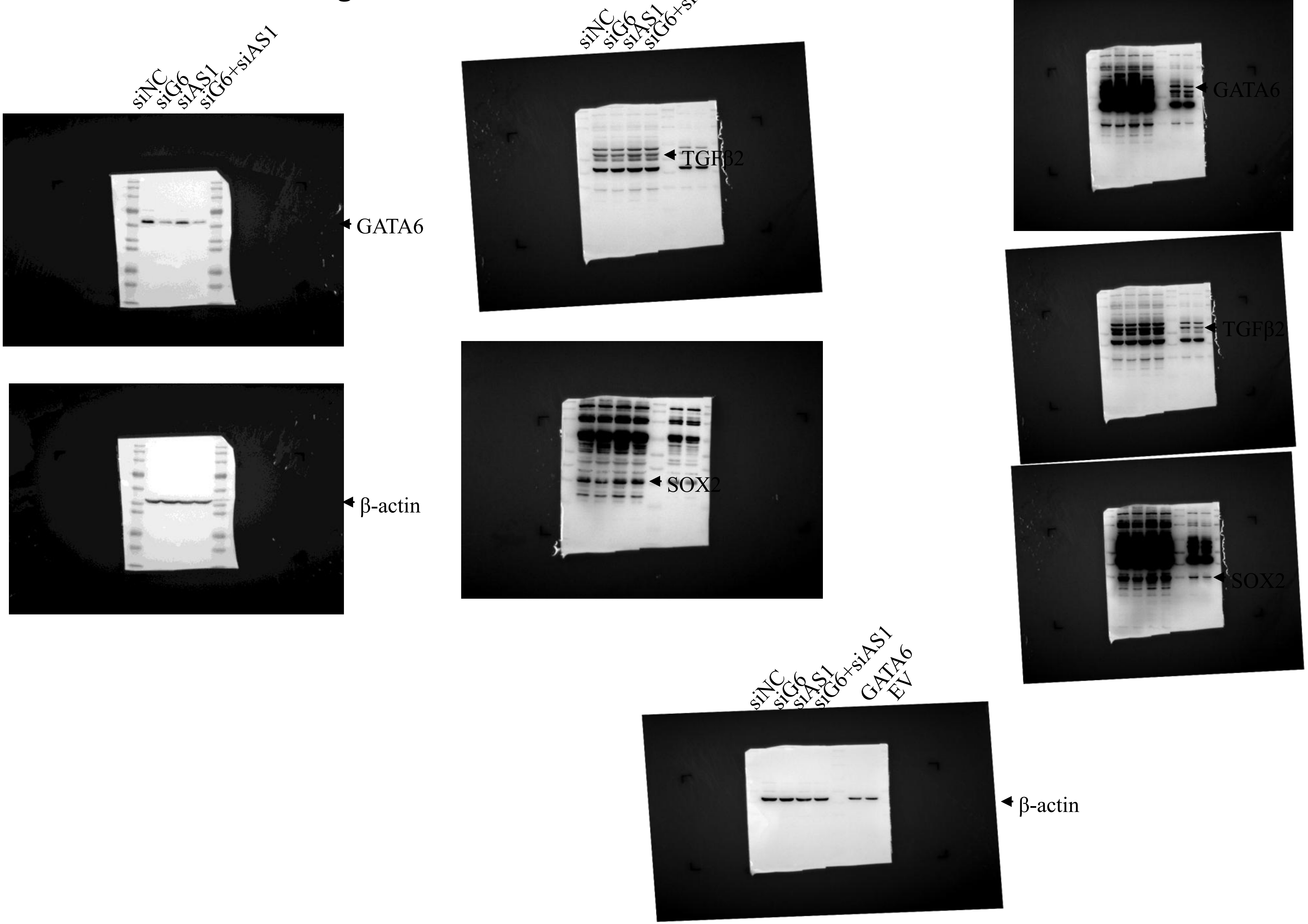
siNC
siG6
siAS1
siG6+siAS1
GATA6
β-actin
TGFβ2
SOX2
GATA6
EV
GATA6
TGFβ2
SOX2
siNC
siG6
siAS1
siG6+siAS1
GATA6
EV
β-actin

Fig. 4C

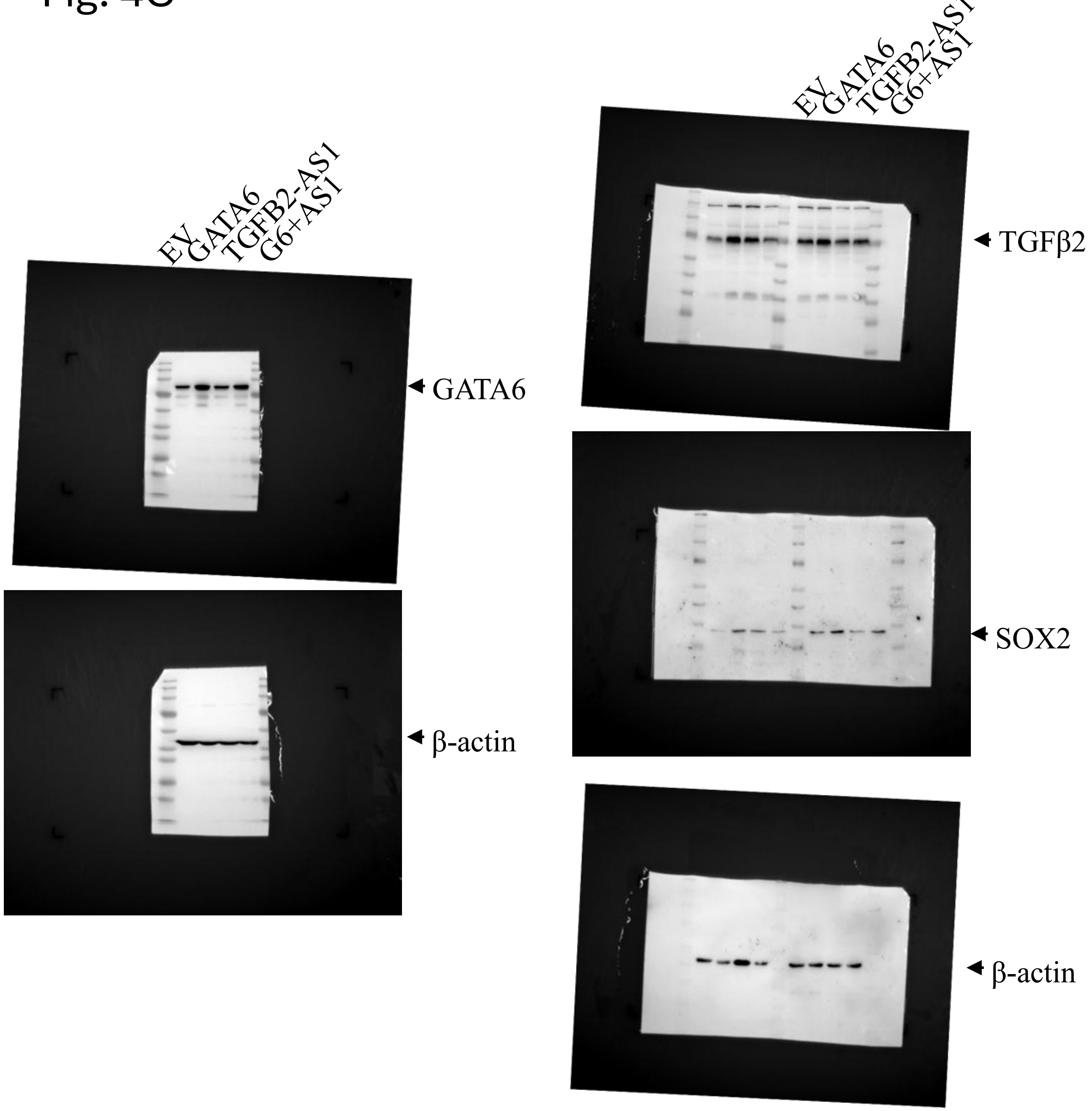

EV
GATA6
TGFB2-AS1
G6+AS1
GATA6
β-actin
EV
GATA6
TGFB2-AS1
G6+AS1
TGFβ2
SOX2
β-actin

Fig. 4D

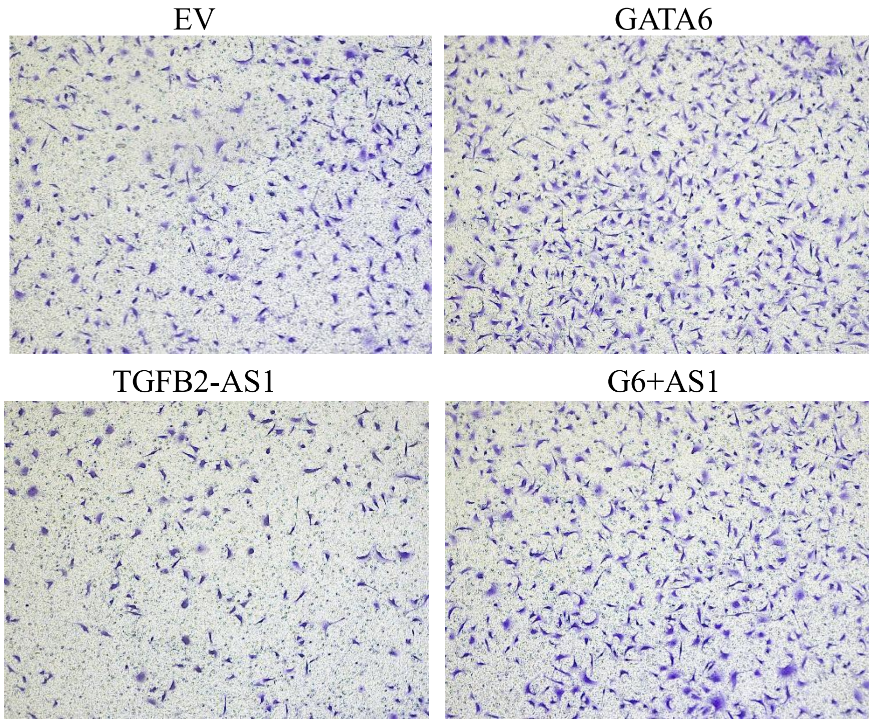
EV
GATA6
TGFB2-AS1
G6+AS1

Fig. 4E

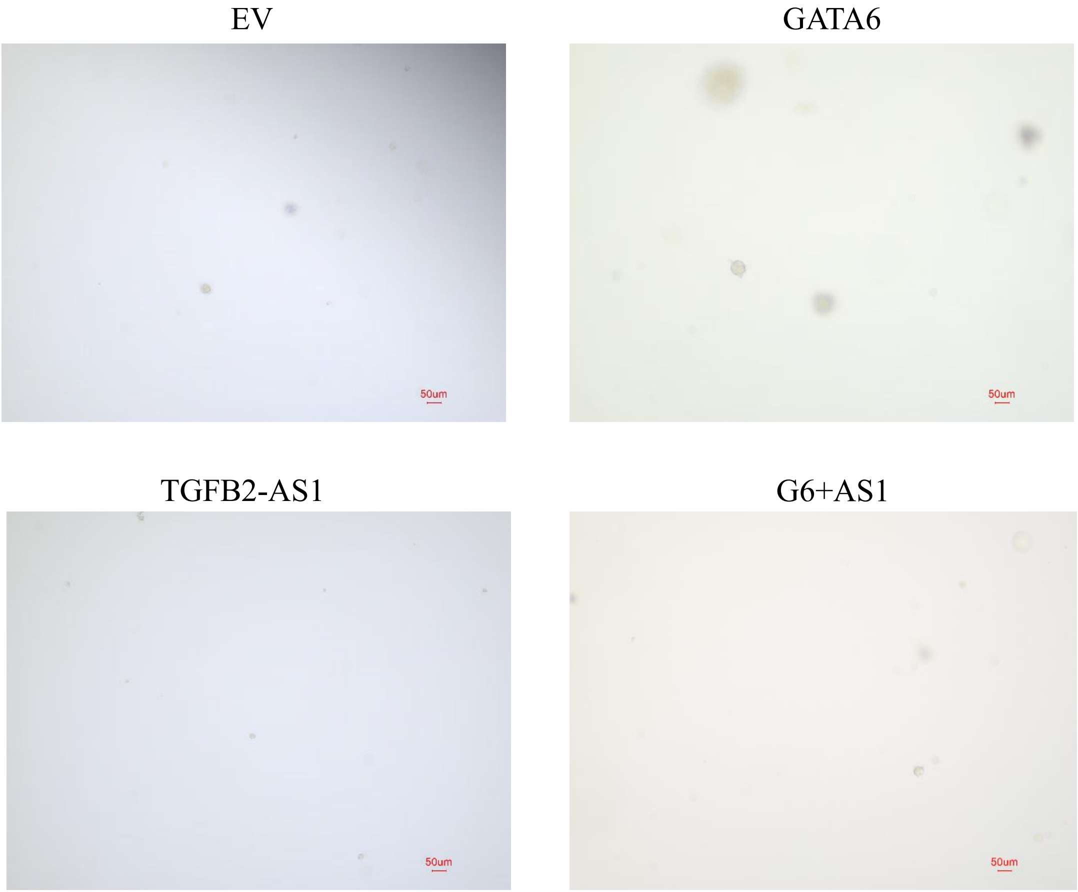
EV
GATA6
TGFB2-AS1
G6+AS1
50um
50um
50um
50um

Fig. 5A

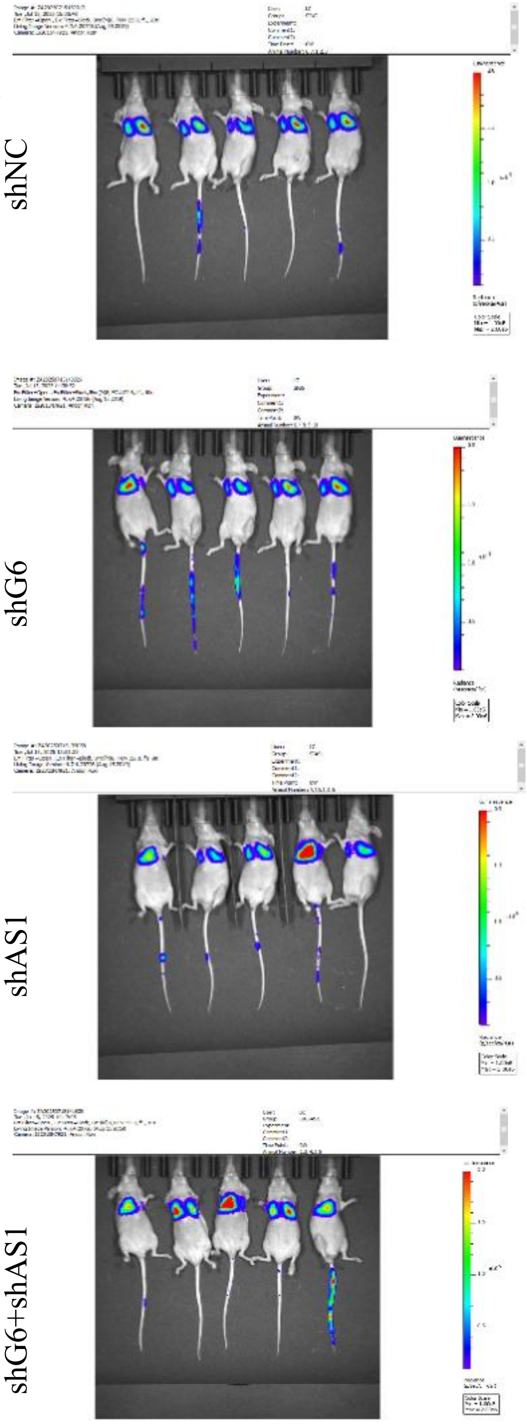

Fig. 5B

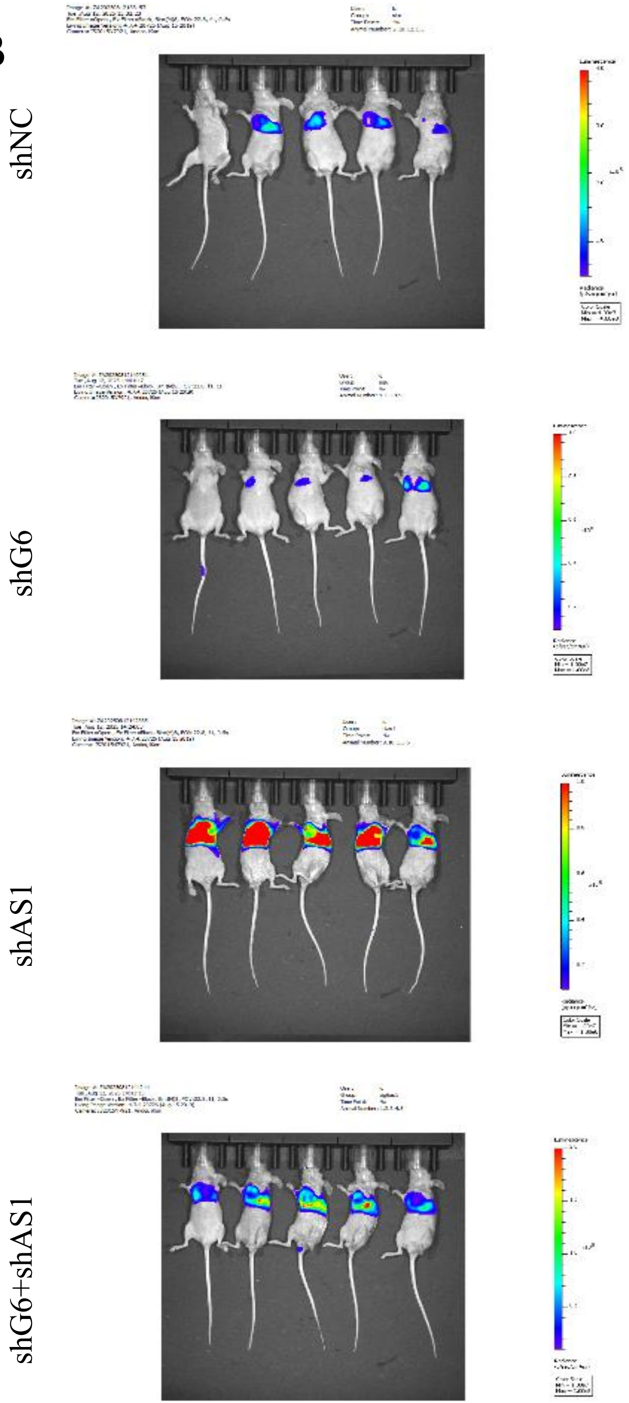

Fig. 5E

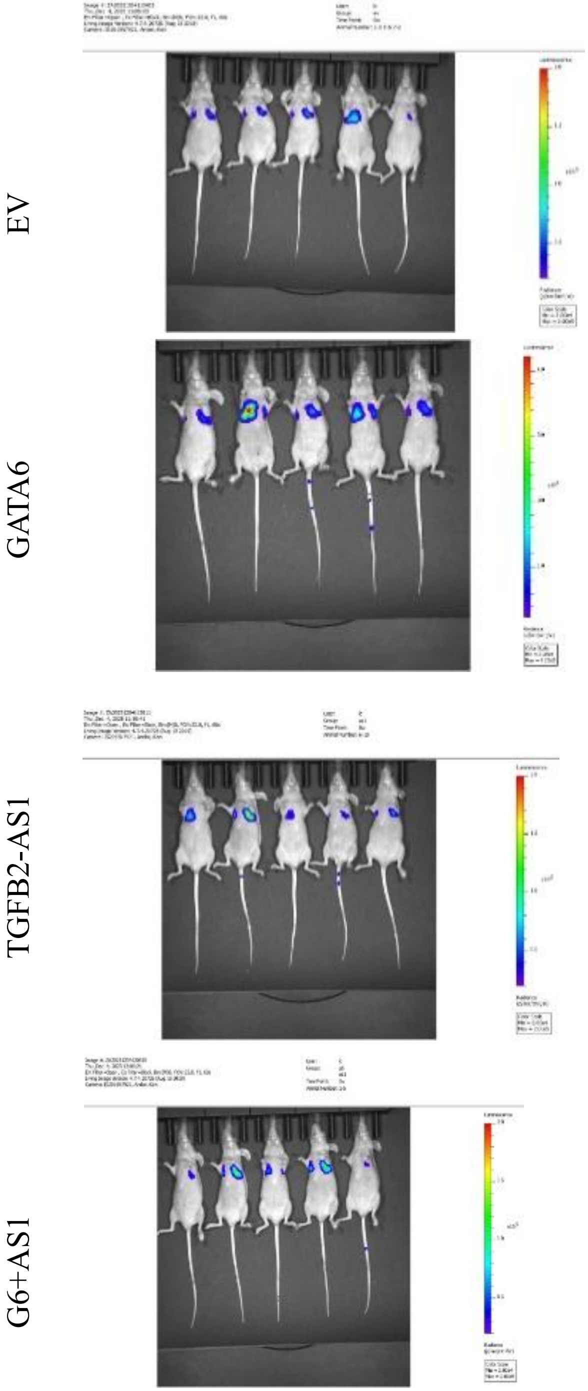

Fig. 5F

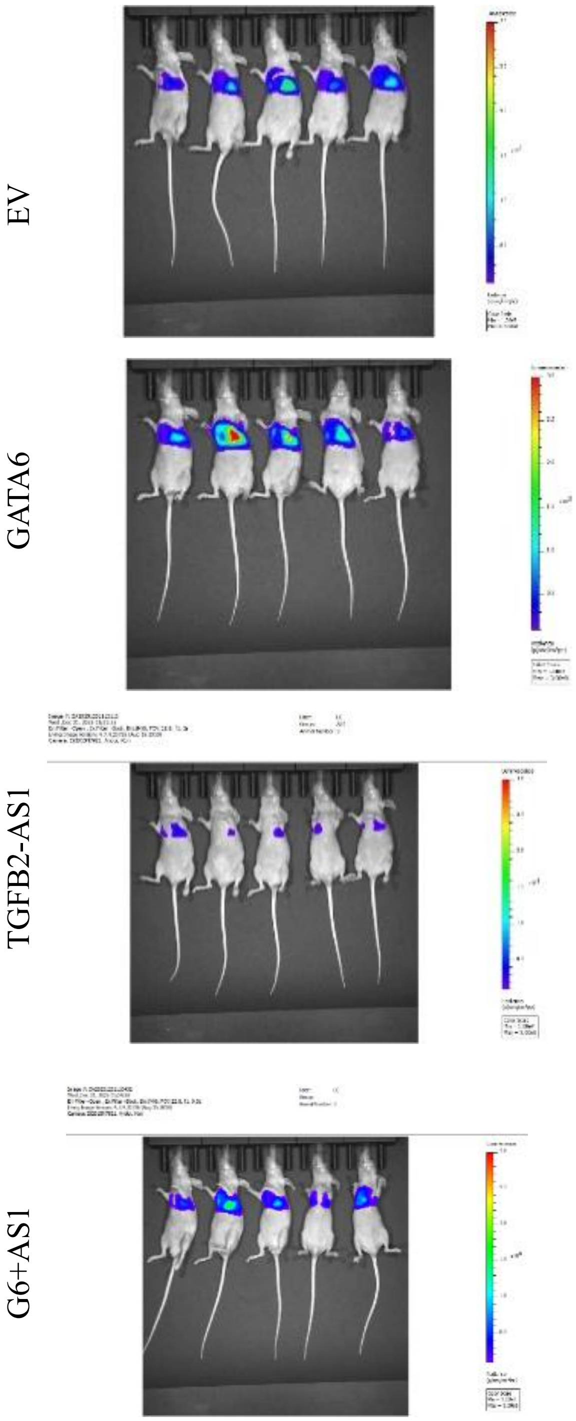

Fig. 5C

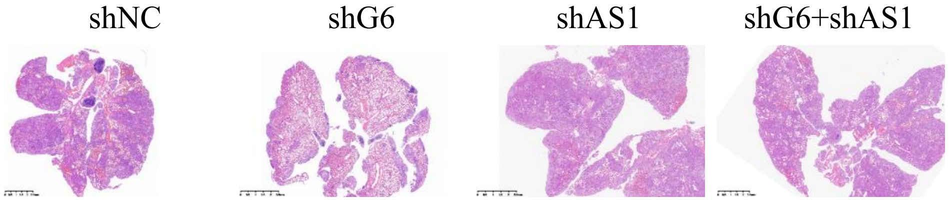

Fig. 5G

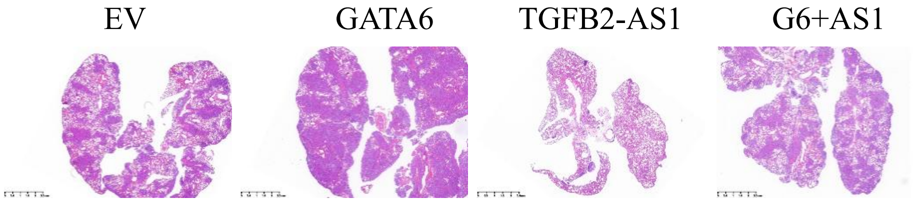

Supplementary Fig. 2C

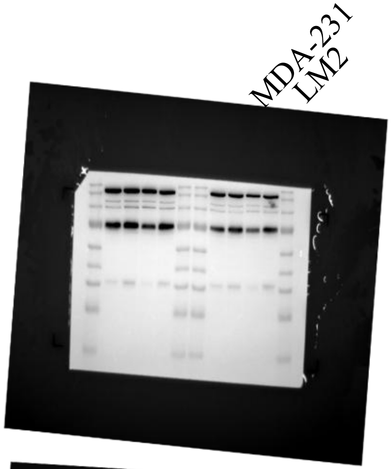

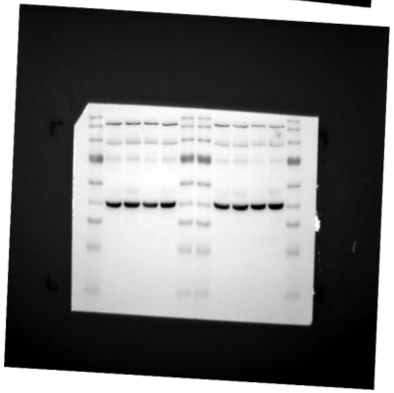

Supplementary Fig. 3A

siNC siG6#1 siG6#2

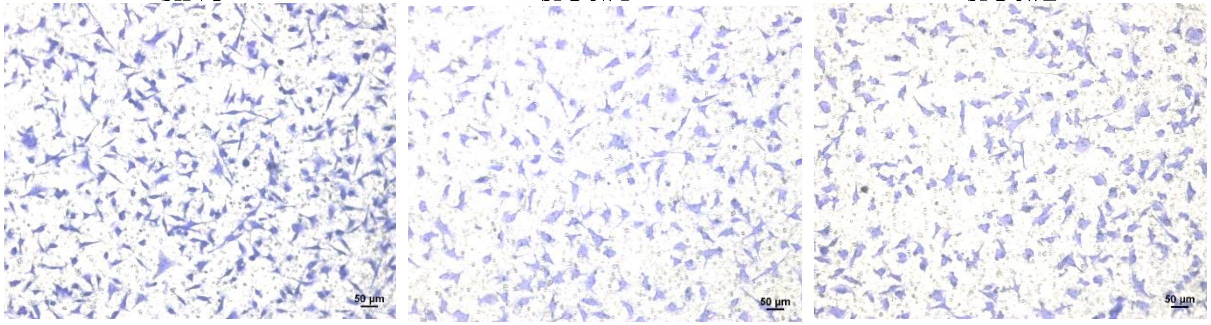

Supplementary Fig. 3B

siNC siG6#1 siG6#2

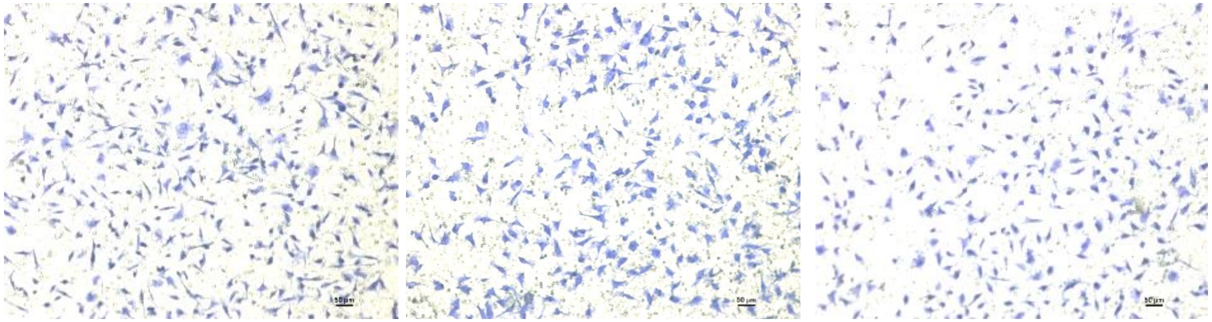

Supplementary Fig. 3D

siNC siG6#1 siG6#2

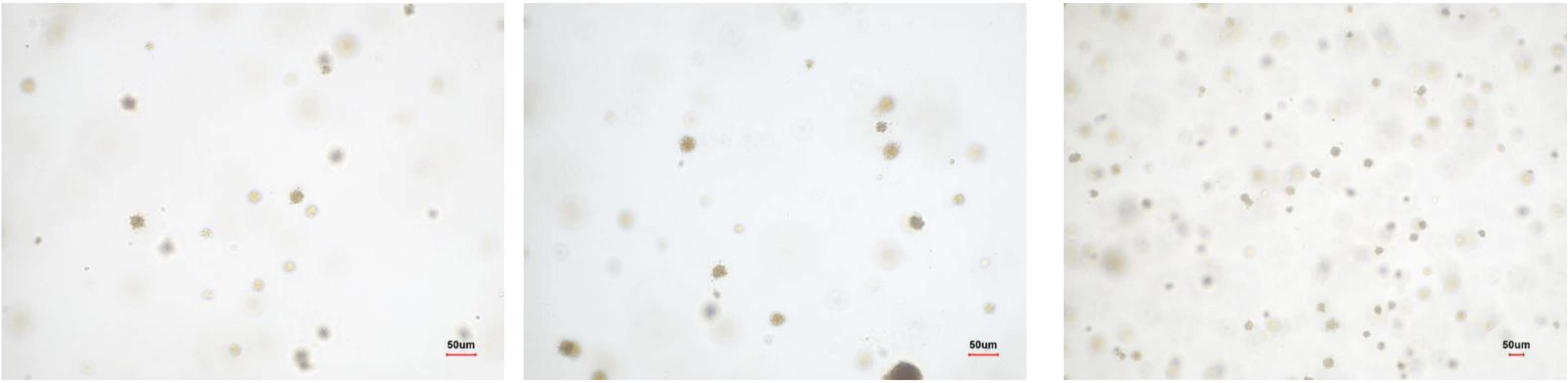

Supplementary Fig. 3C

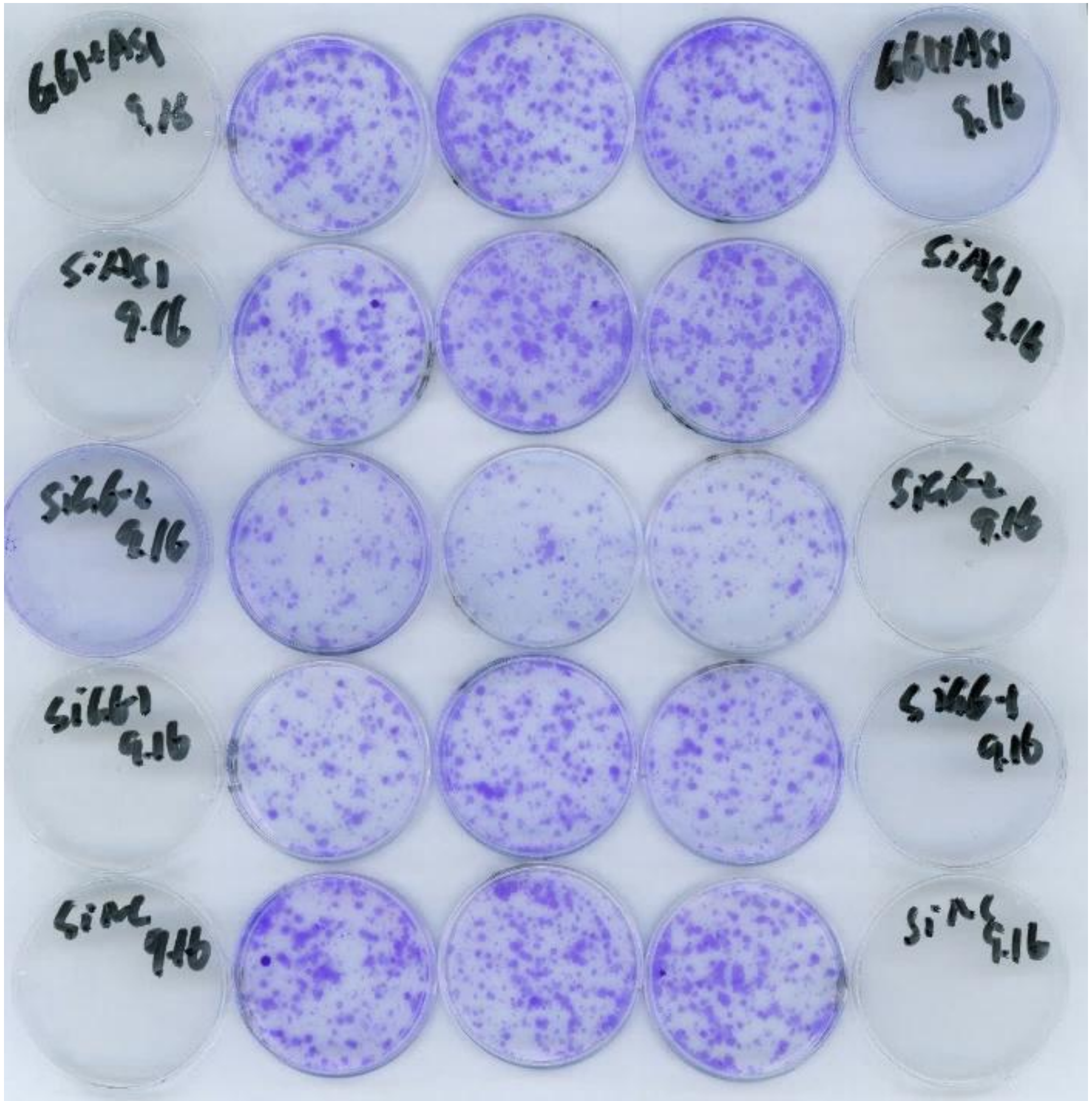

Supplementary Fig. 3F

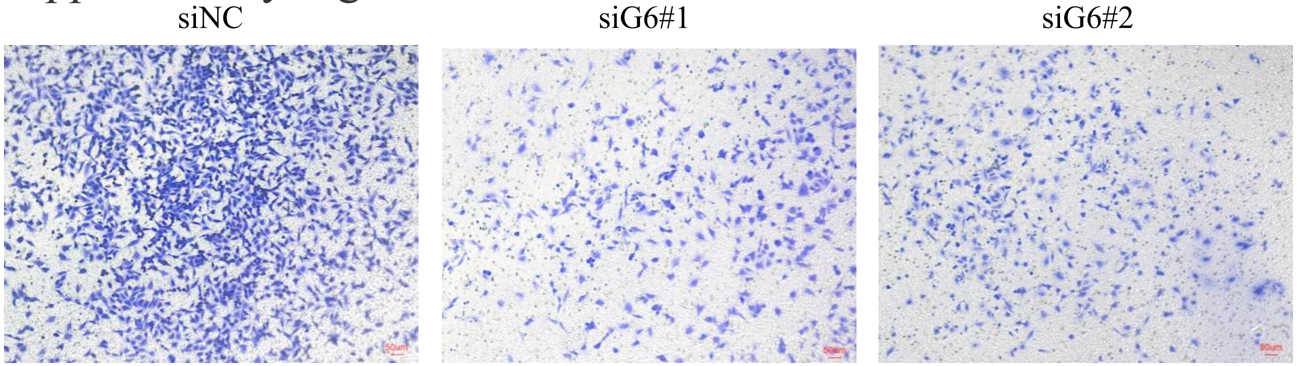

Supplementary Fig. 3G

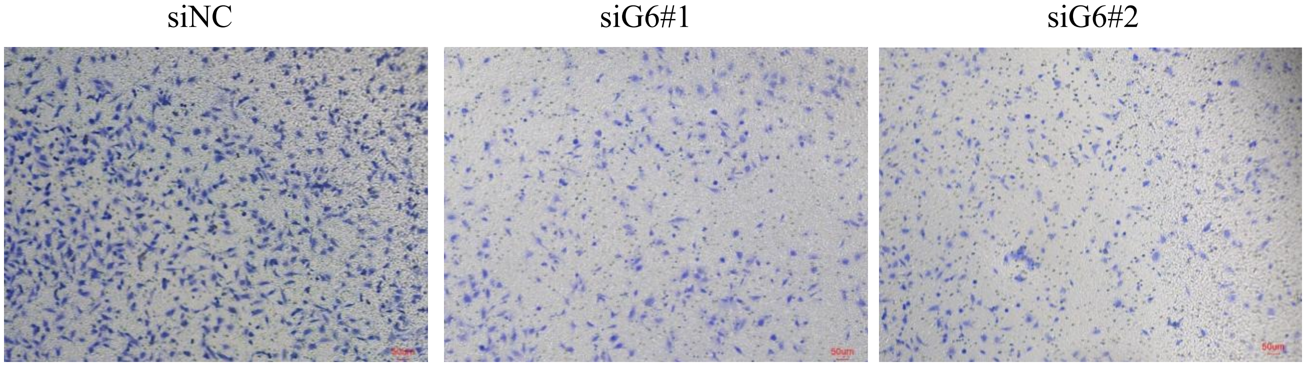

Supplementary Fig. 4H

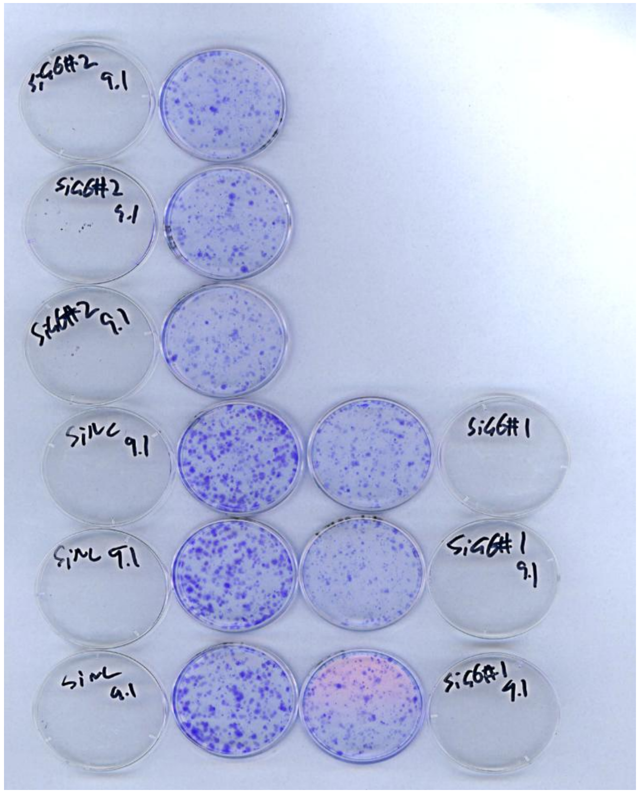

Fig. 4I

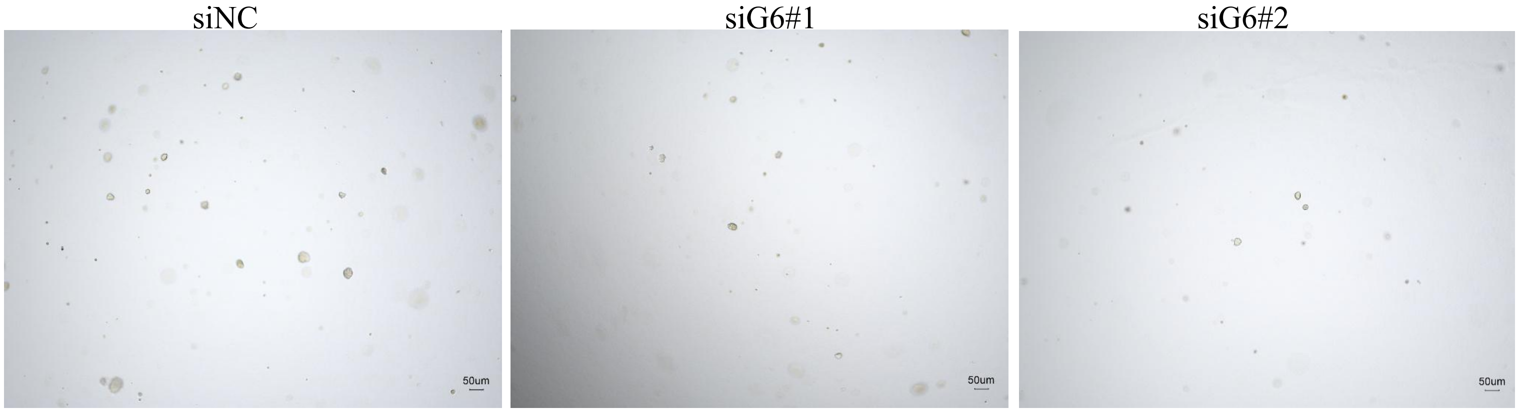

Supplementary Fig. 4D

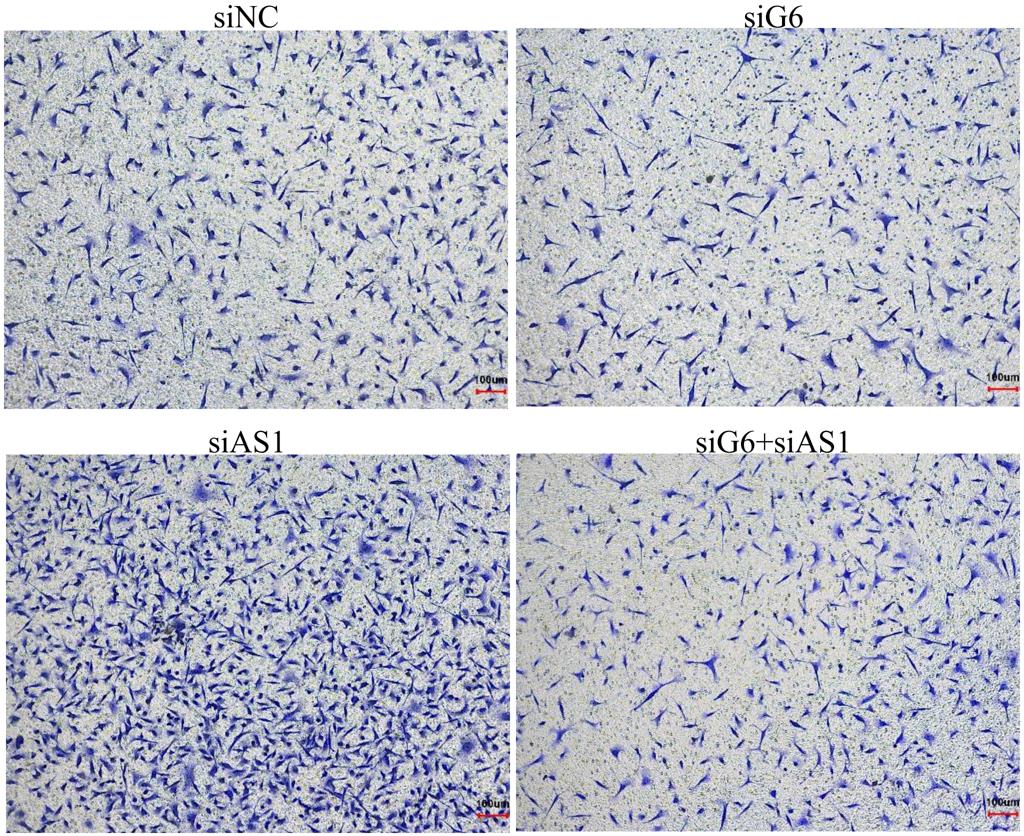

Supplementary Fig. 4E

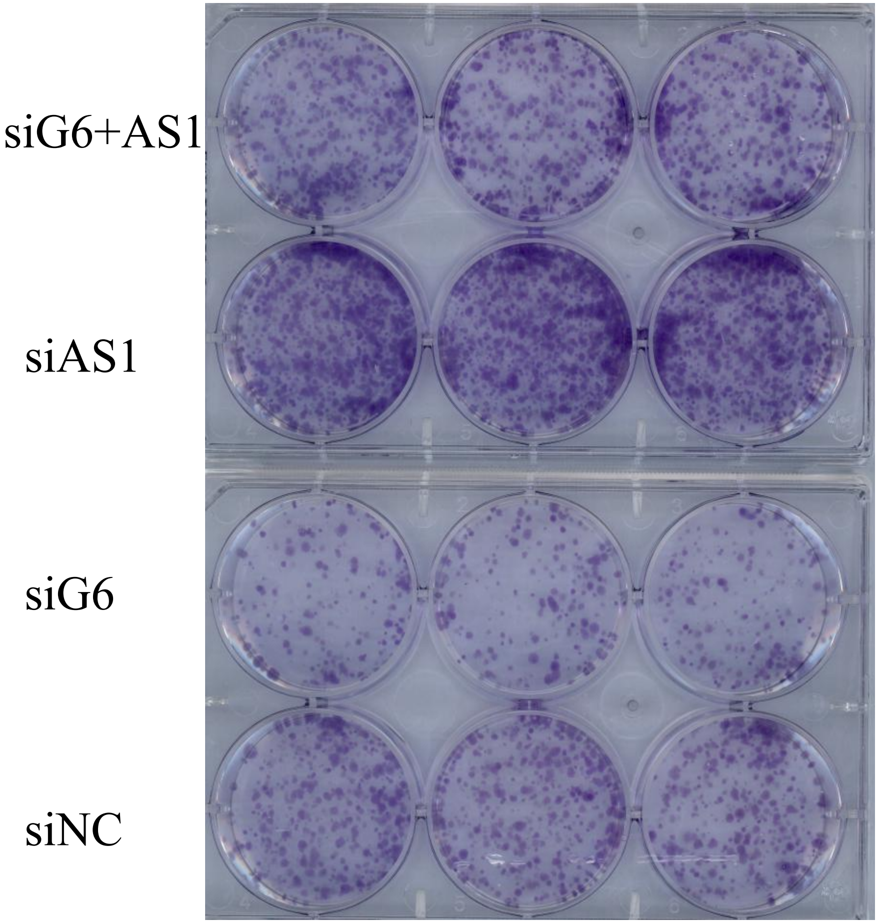

Supplementary Fig. 5B

Supplementary Fig. 5A

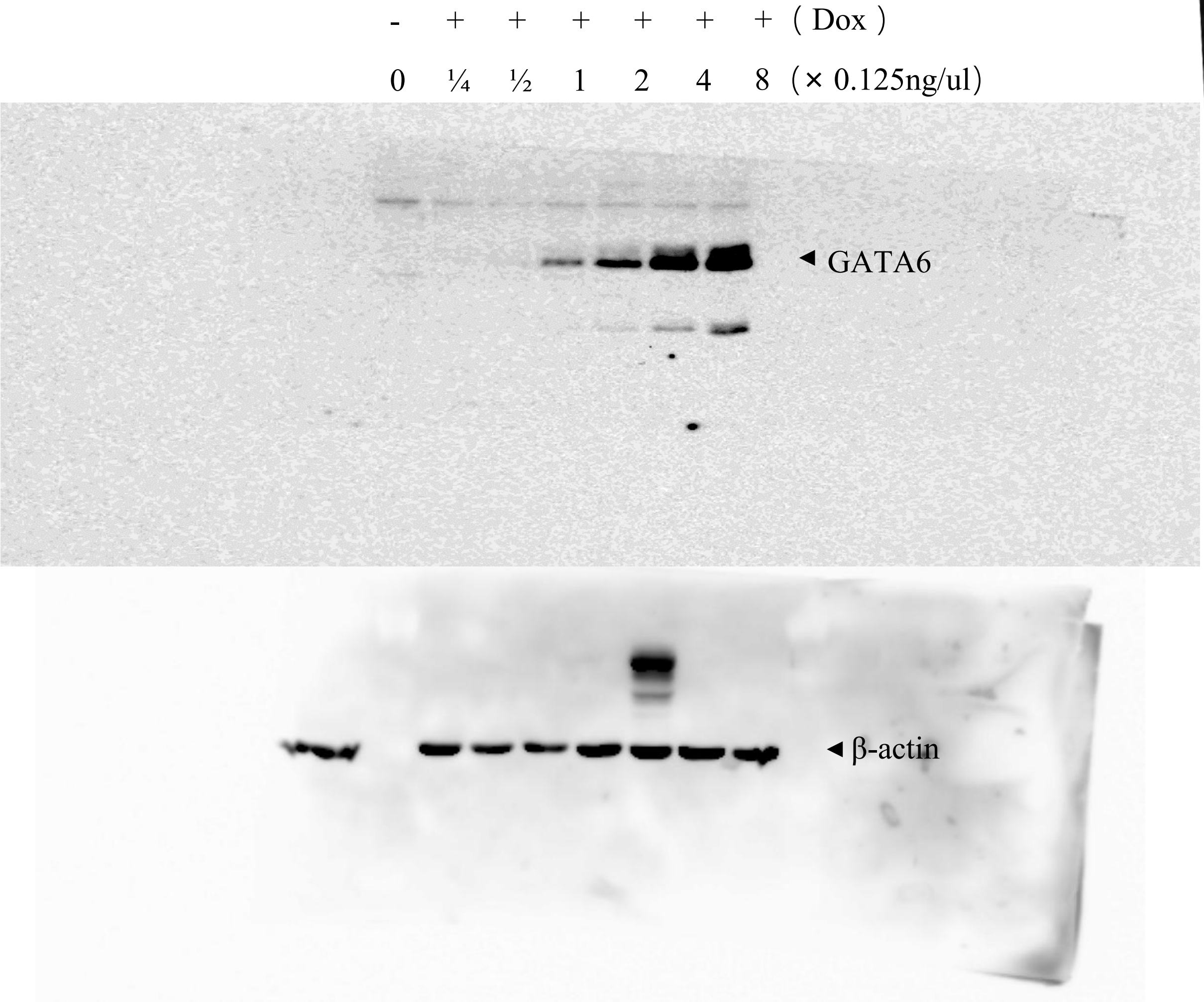

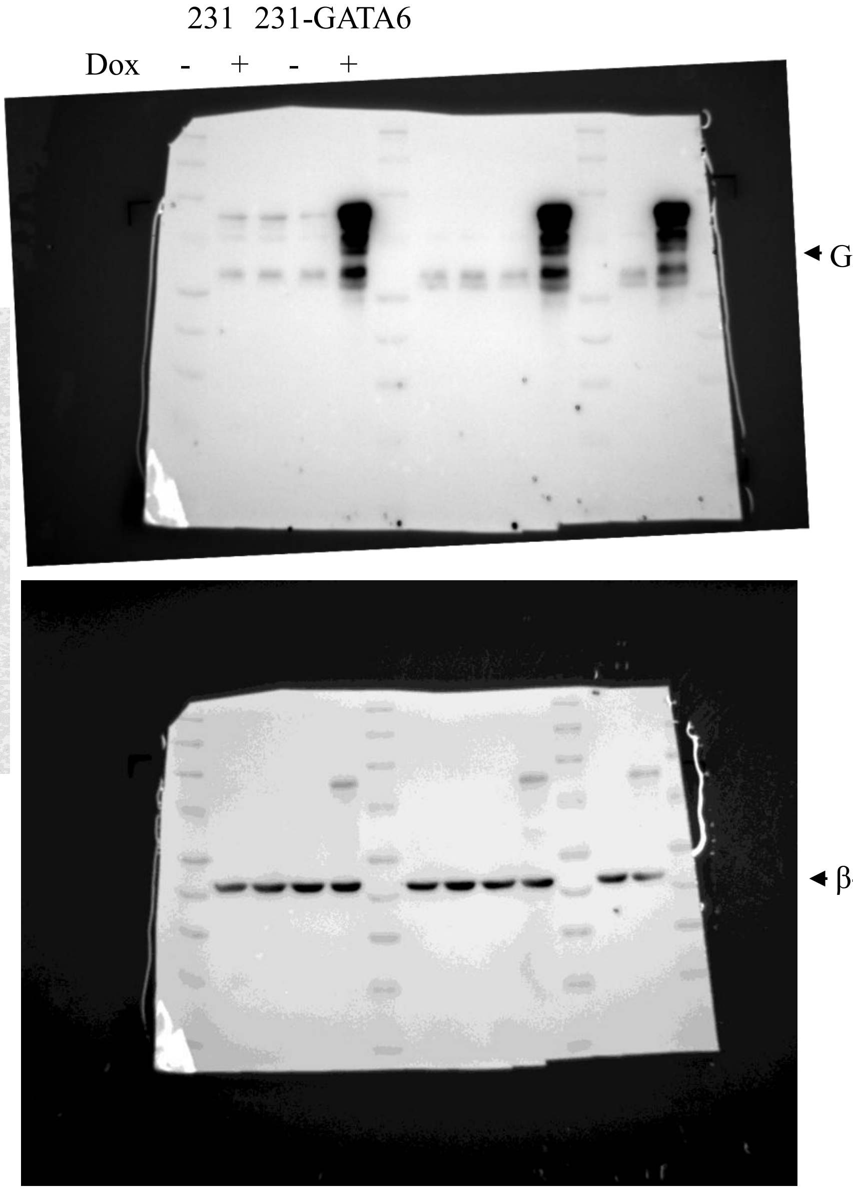

Supplementary Fig. 6A

Dox - Dox+

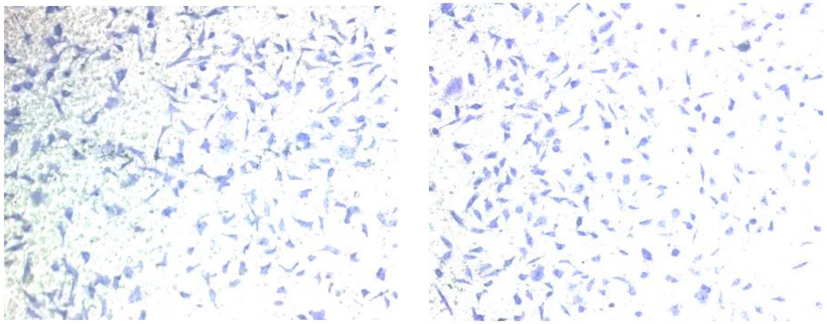

Supplementary Fig. 6C

Dox+

Dox-

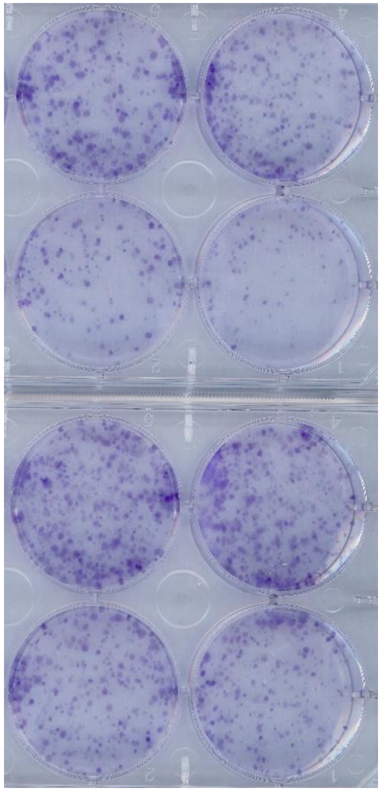

Supplementary Fig. 6B

Dox- Dox+

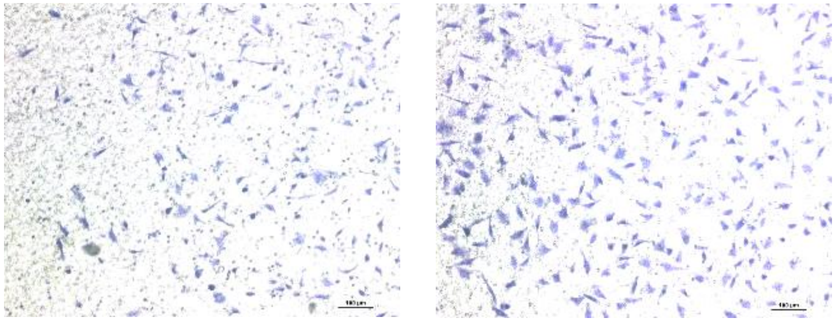

Supplementary Fig. 6D

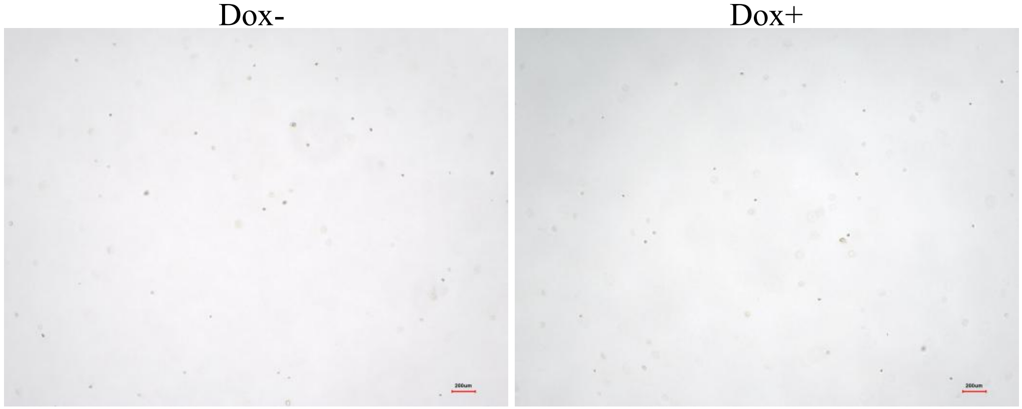

Supplementary Fig. 6F

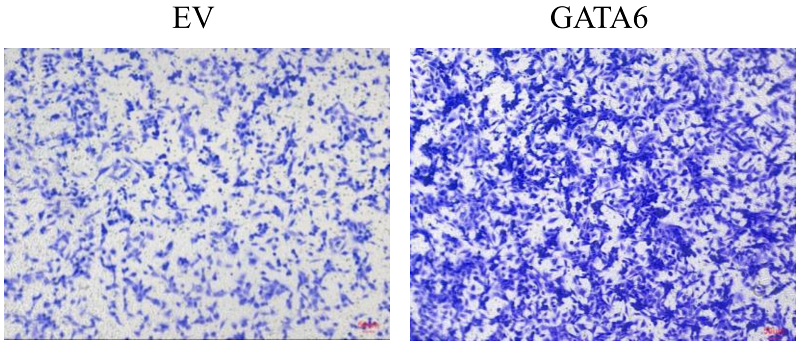

Supplementary Fig. 6G

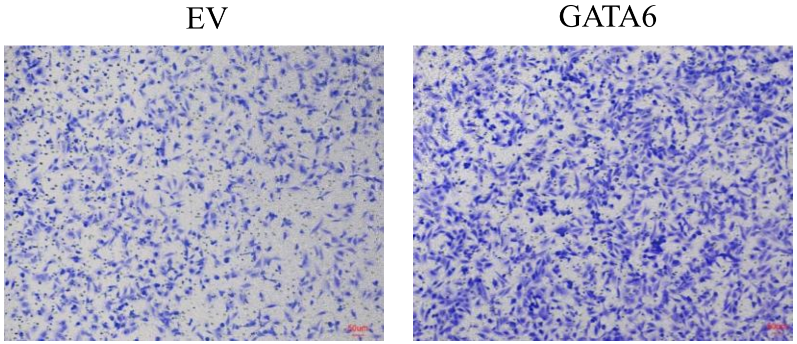

Supplementary Fig. 6H

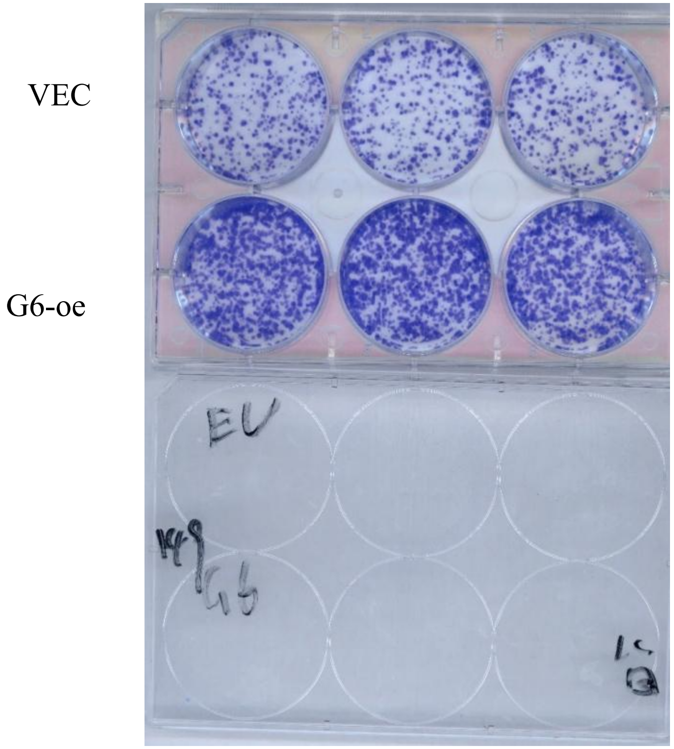

Supplementary Fig. 6I

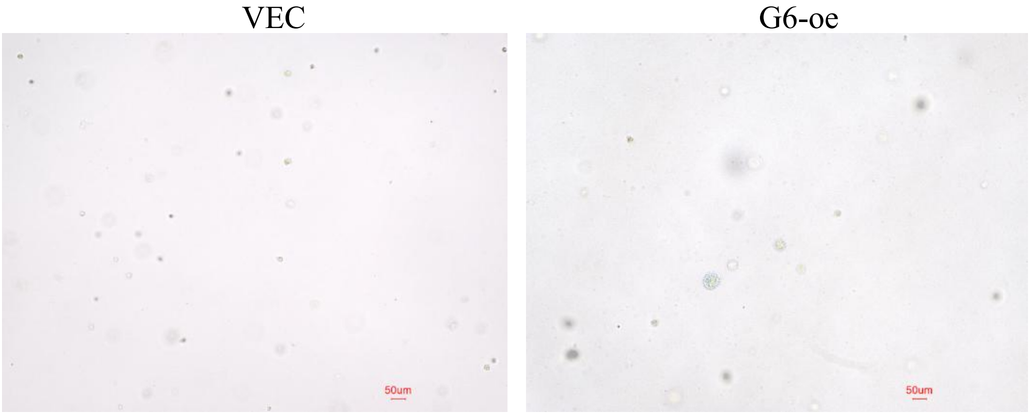

Supplementary Fig. 6K

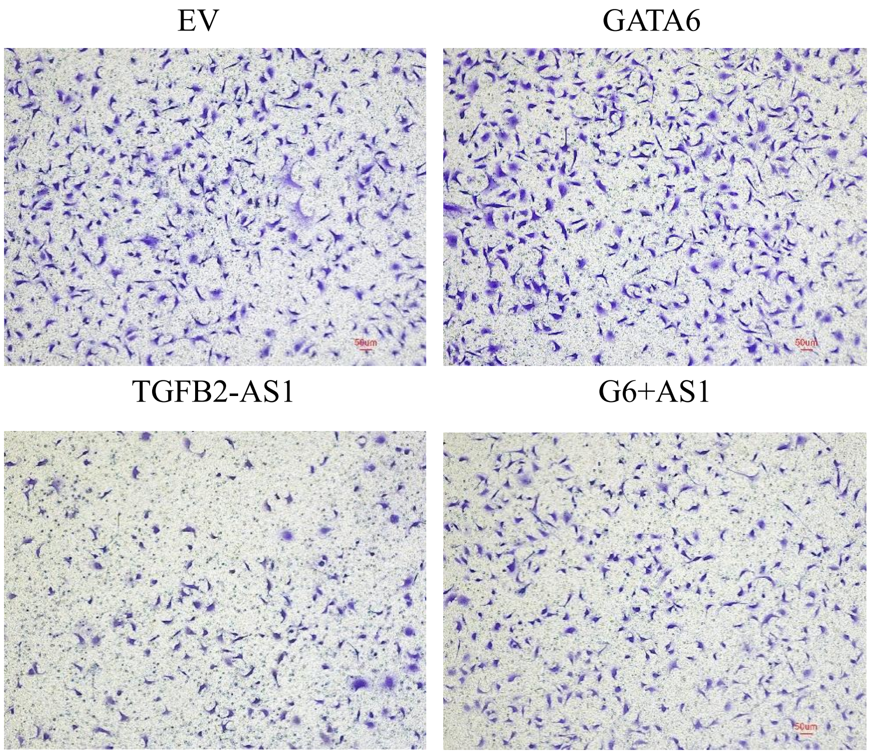

Supplementary Fig. 6L

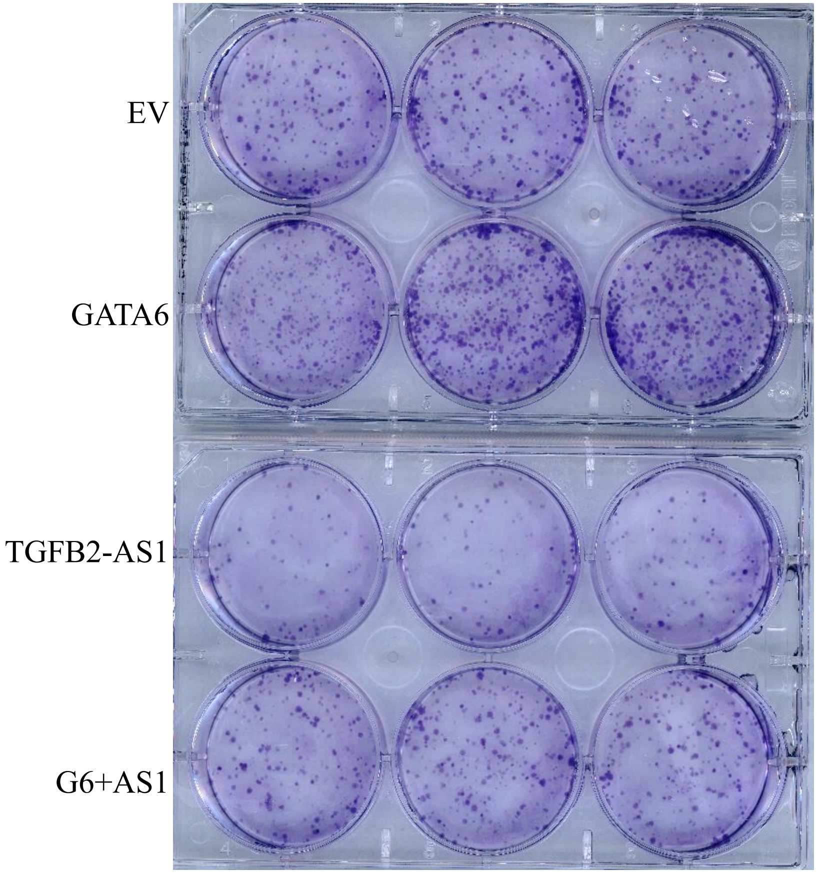

Supplementary Fig. 7C

shNC
shG6
shAS1
shG6+shAS1

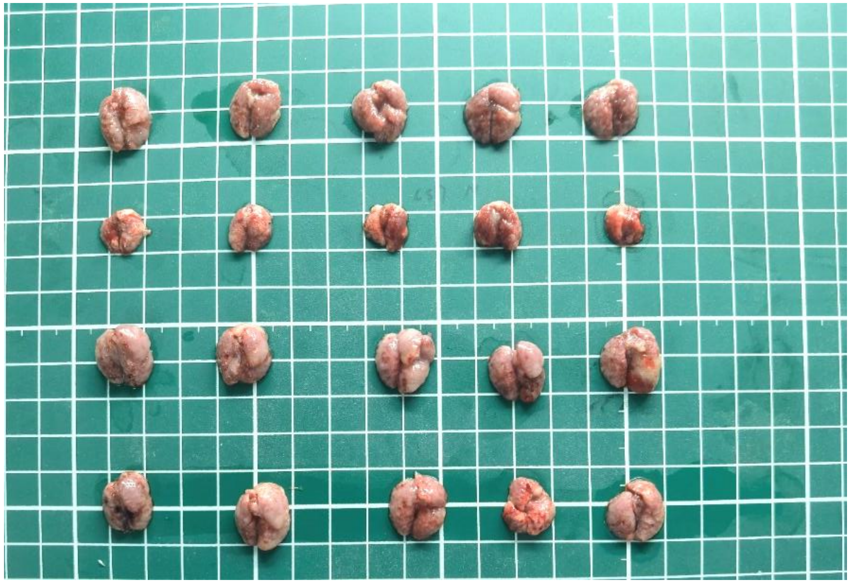

Supplementary Fig. 7F

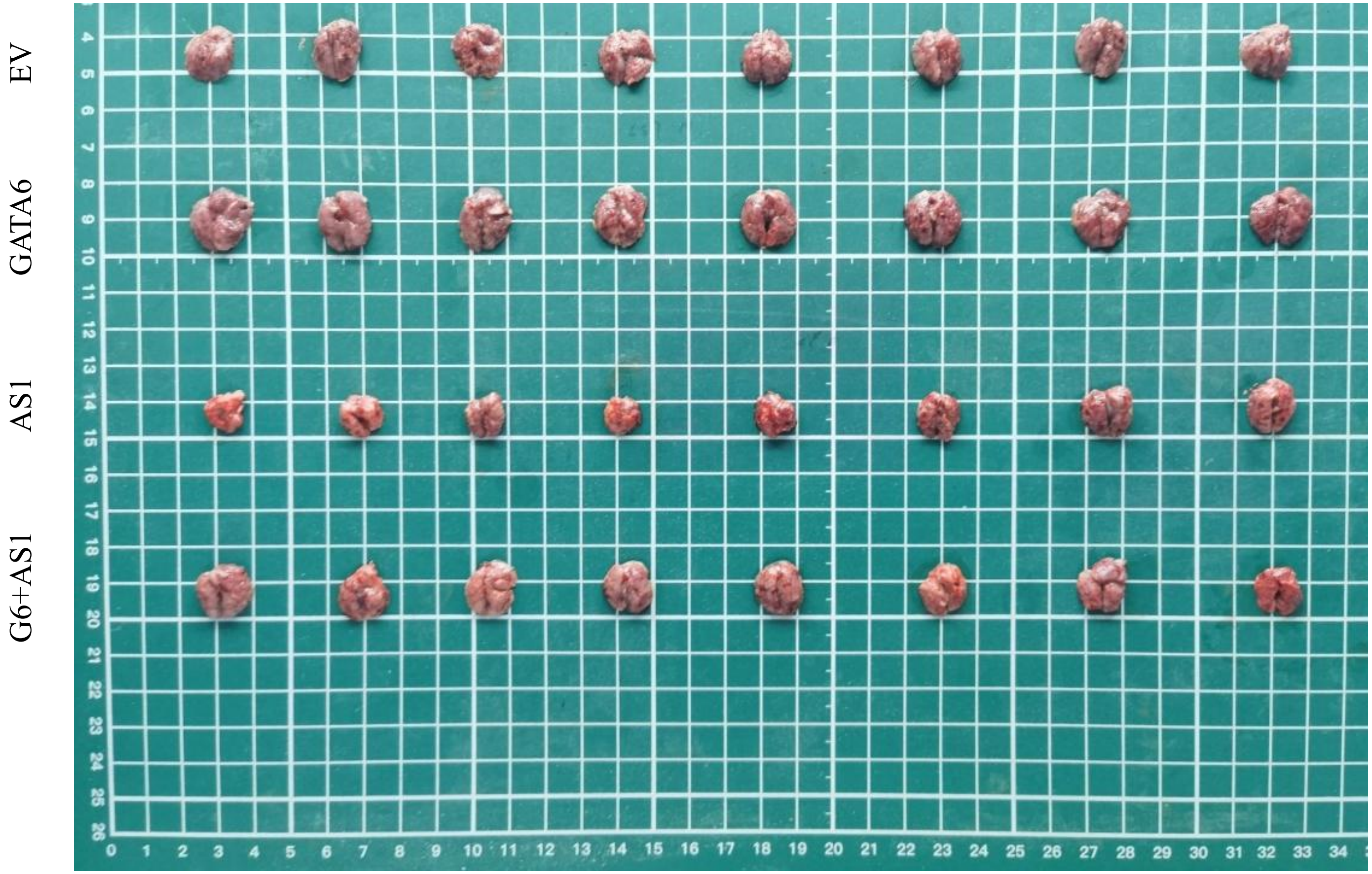

Supplement: Supplementary file 7 — Supplementary Material 7 [file 13402_2026_1195_MOESM7_ESM.zip › 原始结果.pdf]
